# Photoelectron Spectroscopic Determination of the Interfacial Energetics of Metal Oxide Protection Layers on p‑InP Photocathodes

**DOI:** 10.1021/acs.jpcc.5c08203

**Published:** 2026-02-23

**Authors:** Dominic Covelli, Alexandre Z. Ye, Jake M. Evans, Ty A. Schaller, Xinyi Elaine Shen, Paul J. L. Bean, Nathan S. Lewis

**Affiliations:** † Division of Chemistry and Chemical Engineering, 6469 California Institute of Technology, Pasadena, California 91125, United States; ‡ Beckman Institute, 6469 California Institute of Technology, Pasadena, California 91125, United States

## Abstract

The interfacial energetics between p-type InP and a series of metal oxides, including TiO_2_, Nb_2_O_5_, Ta_2_O_5_, and HfO_2_, were evaluated using X-ray photoelectron spectroscopy, ultraviolet photoelectron spectroscopy, and optical absorption spectroscopy. The energy of the conduction band minimum (**E**_cb_) of TiO_2_ and Nb_2_O_5_ was more negative (i.e., further from the vacuum level) than the conduction band minimum at the surface of InP (**E**_cb,s,InP_), whereas **E**_cb_ for Ta_2_O_5_ and HfO_2_ was more positive than **E**_cb,s,InP_. The data are consistent with the electrochemical behavior of p-InP coated with various metal oxide candidate protection layers, with TiO_2_ and Nb_2_O_5_ facilitating interfacial transfer of photogenerated minority-carrier electrons in p-InP photocathodes, and Ta_2_O_5_ and HfO_2_ blocking photogenerated electrons in p-InP from readily transferring across the oxide-coated photocathodes. The energy of the valence band maximum (**E**_vb_) for all of the oxides was much more negative than **E**_vb,s,InP_, consistent with observations that these protection layers effectively block hole transport and consequently suppress oxidative degradation of the underlying p-InP photocathodes.

## Graphical abstract

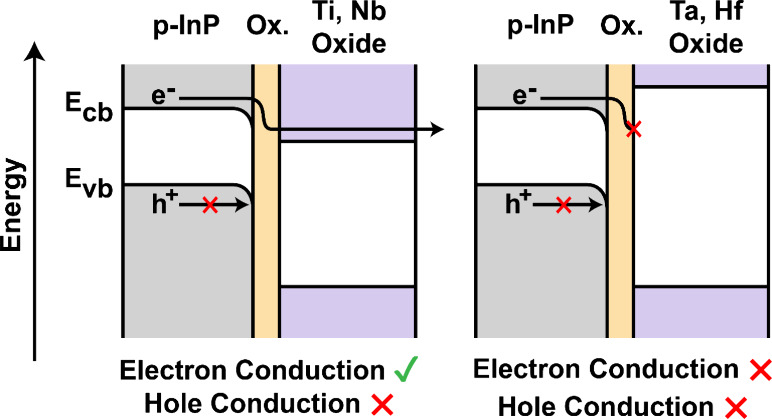

## Introduction

The efficient, sustained production of fuels in photoelectrochemical cells (PECs) requires stable semiconductor photoelectrodes that absorb sunlight effectively.^1^ All semiconductor photoelectrodes are however thermodynamically unstable under illumination in aqueous solution.^2–4^ The stability of many photoelectrodes has been extended by protection layers that provide a barrier against corrosion while maintaining efficient charge transfer between the semiconductor and the electrolyte.^5^ Relatively thick (10–100 nm) titanium dioxide (TiO_2_) protection layers deposited by atomic layer deposition extend the operation of a variety of semiconductor photoanodes for the oxygen-evolution reaction (OER) in aqueous alkaline solutions.^6^ The TiO_2_ layers protect the photoanodes from corrosion while facilitating hole transfer through a defect band in the oxide.^7^ For example, under simulated sunlight, TiO_2_-coated Si microwire photoanodes effect the OER continuously for >2200 h.^8^

The interfacial energetic requirements for optimal protection of photocathodes differ significantly from the energetic requirements for optimal protection of photoanodes. For an InP photocathode, the protection layer must facilitate transfer of photogenerated electrons from the InP conduction band into the solution to drive reduction chemistry, while blocking holes in the InP valence band from reaching the solution, to suppress oxidative degradation pathways at the photocathode surface and to limit charge-carrier recombination.^9,10^ Consequently, the energy of the conduction band minimum (**E**_cb_) of the protection layer should be closely aligned with, or slightly more negative in energy (i.e., further from the vacuum level) than the conduction band minimum at the surface of the InP (**E**_cb,s,InP_), to preclude formation of an energetic barrier to electron conduction from the InP into the solution. Moreover, to effectively drive the hydrogen-evolution reaction (HER), **E**_cb_/*q* of the protection layer must be more negative than the reversible potential of the hydrogen electrode,^1^ RHE, in the electrolyte of interest.

Nb_2_O_5_ has been recently shown to produce a beneficial protection layer for platinized p-InP photocathodes performing the HER in acidic aqueous electrolytes, permitting photocathodic current but blocking dark anodic current, whereas HfO_2_ and Ta_2_O_5_ block both photocathodic current and dark anodic current at p-InP | metal oxide | Pt | H_2_SO_4_(aq) interfaces.^11^

To elucidate the fundamental factors that control the interfacial energetic behavior, the interfacial energetics of heterojunctions between p-InP and metal oxide protection layer candidates including TiO_2_, Nb_2_O_5_, Ta_2_O_5_, and HfO_2_ have been determined herein using a combination of X-ray photoelectron spectroscopy (XPS) and ultraviolet photoelectron spectroscopy (UPS). XPS allows measurement of the composition and relative band edge positions of the interfacial layers, whereas UPS allows measurement of the work function and the difference in energy between **E**_vb_ and the Fermi level (**E**_F_) of the sample, enabling the identification of interfacial dipoles that influence charge transfer.^7^ These measurements have facilitated construction of band diagrams that provide guidance for selecting suitable protection layers for p-InP photocathodes effecting the HER.

## Experimental Methods

### Preparation of InP Substrate

Single-side polished p-type indium phosphide wafers (Zn-doped, with a dopant density *N*_A_ = 1–8 × 10^17^ cm^–3^, AXT Inc.) with a (100) orientation were used as substrates for atomic layer deposition. Ohmic back contacts to p-InP were made by RF sputtering under 2 mTorr of Ar 10 nm of Zn (at 60 W of power) followed by 90 nm of Au (at 70 W of power) onto the back (unpolished) side of the InP wafers. The contacts were then annealed under forming gas for 10 min at 400 °C. Small (<1 cm^2^) pieces of p-InP with ohmic back contacts were cut from the wafer using a scribe. The pieces were etched by immersion for 15 s in a solution of 8 μL Br_2_ (reagent grade, Sigma-Aldrich) in 20 mL methanol (anhydrous, 99.8%, Sigma-Aldrich). The etched samples were rinsed with methanol, immersed for 15 s in a solution of 1.12 g of KOH (pellets, Macron Fine Chemicals) in 20 mL of deionized H_2_O, and subsequently rinsed again with methanol. This entire process was immediately repeated so that in total, the InP was immersed twice in the Br_2_ solution and twice in the KOH­(aq) solution. The samples were then blown dry under a stream of N_2_(g).

### Atomic Layer Deposition of Metal Oxides onto InP Samples

Metal oxide films were deposited onto InP using a Cambridge Nanotech S200 ALD system. After etching, InP wafers were immediately introduced into the ALD chamber (<60 s exposure to air). Each ALD cycle consisted of a pulse of the metal-oxide precursor followed by a pulse of deionized water (Table 1). Between each pulse the chamber was purged with a constant 0.02 L min^–1^ flow of ultra high purity N_2_(g). When idle, the ALD system was maintained under a continuous N_2_(g) purge and had a background pressure of ∼2.0 × 10^–1^ Torr.

**Table 1:** Atomic Layer Deposition Recipes for Various Protection Layer Candidates

Precursor identity	TDMAT^a^ (Ti)	PDMAT^b^ (Ta)	TBTDEN^c^ (Nb)	TDMAH^d^ (Hf)
Inner heater temperature (°C)	150	150	250	150
Outer heater temperature (°C)	150	150	190	150
Manifold temperature (°C)	150	150	150	150
Precursor temperature (°C)	90	120	120	90
Water temperature (°C)	no heating (room temp.)	no heating (room temp.)	no heating (room temp.)	no heating (room temp.)
Precursor pulse duration (s)	0.15	0.15	0.2	0.15
Purge duration following precursor pulse (s)	15	15	15	15
Water pulse duration (s)	0.015	0.015	0.015	0.015
Purge duration following water pulse (s)	15	60	60	15

a Tetrakis­(dimethylamido)­titanium­(IV) (99.999% trace metals basis, Sigma-Aldrich).

b Pentakis­(dimethylamino)­tantalum­(V) (98%+, Strem Chemicals, Inc.).

c (*t*-Butylimido)­tris­(diethylamino)­niobium­(V) (98%+, Strem Chemicals, Inc.).

d Tetrakis­(dimethylamino)­hafnium­(IV) (98%+, Strem Chemicals, Inc.)

Samples for photoelectron spectroscopy were prepared using 50 ALD cycles of the specified metal oxide, except for HfO_2_-coated InP, which was deposited using only 20 ALD cycles to minimize obscuration of the underlying InP signals. For UV–Vis spectroscopy, clean quartz substrates were coated with 400 ALD cycles of the corresponding oxide.

### Photoelectron Spectroscopy Measurements

X-ray photoelectron spectroscopy (XPS) was performed using a Kratos Axis Ultra spectrometer (Kratos Analytical, Manchester, UK) with a base pressure of <3 × 10^–9^ Torr. The instrument was equipped with a hybrid magnetic and electrostatic electron lens system, a delay-line detector (DLD), and a monochromatic Al Kα X-ray source (1486.6 eV). Except during angle-resolved measurements, the hemispherical analyzer was oriented for detection along the sample surface normal to maximize the depth sensitivity of the measurement. The instrument was operated using Vision Manager software v2.2.10 revision 5, and XPS data were analyzed using CasaXPS software v2.3.25PR1.0 (CASA Software Ltd.).

Ultraviolet photoelectron spectroscopy (UPS) was performed in the same Kratos Axis Ultra system. Using a He discharge lamp that was attached to the analysis chamber, the samples were irradiated with 21.2 eV photons from the Helium I emission line.

After ALD, samples were immediately transferred to the load lock of the Kratos spectrometer and held under vacuum (<5 min exposure to air). p-InP substrates without ALD oxide films were etched and immediately transferred to the load lock and held under vacuum (<1 min exposure to air). Five spectra for each energy region were collected from different locations on the sample. The values reported in this manuscript represent averages for the binding energy, work function, and electron takeoff, respectively, for each energy region of each sample.

Additional methodological information is provided in the Supporting Information. Section S1 details the instrument settings and peak fitting constraints. Section S2 describes the calculation of uncertainty values. Section S3 discusses the use of the carbon 1s signal as an internal reference. All XPS and UPS spectra acquired in this study are compiled in Section S4.

### Optical Absorbance Measurements

Optical absorbance measurements were performed using a Cary 5000 UV–Vis–NIR spectrophotometer (Agilent Technologies) equipped with an external DRA 1800 diffuse reflectance attachment. Samples of each metal oxide on clean quartz microscope slides were prepared using 400 atomic layer deposition cycles, with the same ALD recipe as was used for InP. Prior to data acquisition, zero and baseline measurements were recorded. The baseline measurement was established using a clean, unaltered quartz microscope slide. The Cary WinUV software automatically performed zero and baseline corrections on the recorded data. Samples were positioned at the rear port of the integrating sphere, and optical reflection data were normalized to a reflectance standard (99%, LabSphere, Inc.) that was placed in a nominally identical position as the samples. The optical absorption properties were quantified using the Kubelka–Munk (KM) function, which yields a quantity proportional to the absorption coefficient (α) from diffuse reflectance measurements.^12,13^ Reflectance data were first converted from percentage to decimal form. The decimal reflectance (*R*) is given by

$$R=\frac{R_{\%}}{100}$$ 1

The KM function was then calculated as

$$F(R)=\frac{(1-R{)}^{2}}{2R}$$ 2

Tauc plots were generated by plotting (*F*(*R*)·*h*ν)^*n*^ as a function of photon energy (*h*ν), where *n* = 1/2 for indirect band gap semiconductors.^14^

### Determination of Fermi Level Using Dopant Density

All energies were negative relative to the vacuum level, which by definition had an energy of 0 eV. Nernst potentials, *E*, were related to the energies of the semiconductor bands, **E**, using the definition *E* = **E**/*q* where *q* is the (signed) charge on an electron. The energy of the Fermi level (**E**_F_) relative to the energy of the InP valence band maximum (**E**_vb,InP_) was determined using eq 3:

$$E_{F}=E_{\mathrm{vb},\mathrm{InP}}+kT\ln\left(\frac{N_{v}}{p}\right)$$ 3

where *N*_v_ is the effective density of states in the valence band, and *p* is the concentration of holes. The literature value of *N*_v_ for InP is 1.1 × 10^19^ cm^–3^.^15^ In a moderate-to-highly doped semiconductor at room temperature, *p* is approximately equal to the dopant density (*N*_A_). The dopant density was provided by the manufacturer as 1–8 × 10^17^ cm^–3^, so the mean value of 4.5 × 10^17^ cm^–3^ was used as *p* in eq 3. Thus, at *T* = 293 K, **E**_F_ = **E**_vb,InP_ + 0.0807, indicating that the Fermi level is ∼0.0807 eV more positive than **E**_vb,InP_. This value was truncated in this work to two decimal places (0.08 eV) for calculations of all energies in the semiconductor.

### Calculations of Band Diagrams

Section S5 contains information on the construction of band diagrams. Figure S7 provides a schematic representation of the electrode structure. Band diagrams were constructed via stepwise analysis of the different layers and interfaces of interest (Figure S8). Values in the band diagrams for bulk InP were determined from literature sources and calculations. Values in the band diagrams for the InP surface, oxide layer, and protection layer were mostly determined from experimental results, such as from XPS or UPS spectra. Figure S9 provides an annotated band diagram describing the source (XPS, UPS, etc.) of each value in the diagram. In the band diagrams, extracting values for the band bending (**E**_bb_) and the interface dipole (δ) required use of both experimental results and literature values.

**E**_bb_ was calculated as the energy difference between the experimentally determined surface In 4d_5/2_ binding energy and the literature-derived bulk In 4d_5/2_ binding energy (eq 4).

$$E_{\mathrm{bb}}=(\mathrm{In}4d_{5/2}\mathrm{BE}{)}_{\mathrm{surface}}-(\mathrm{In}4d_{5/2}\mathrm{BE}{)}_{\mathrm{bulk}}$$ 4

The work function (ϕ) of a sample was calculated as the difference between the energy of a helium I photon (21.2 eV) and the secondary electron cutoff (SECO) of the UPS data (eq 5):

$$\begin{array}{c} \phi_{\mathrm{Sample}}=21.2-\mathrm{SECO} \end{array}$$ 5

The dipole (δ) at the interface between two semiconductors with different work functions was calculated using eq 6.^7^

$$\begin{array}{c} \delta =\phi_{\mathrm{InP}}-(\phi_{\mathrm{Overlayer}}+E_{\mathrm{bb}}) \end{array}$$ 6

with a positive dipole indicating that the vacuum level of the InP is at a more positive energy than the vacuum level of the contacting oxide.

The energy of an electron in the conduction band at the surface of the p-InP (**E**_cb,s,InP_) relative to the Fermi level was calculated by subtracting the band bending and the energy difference between **E**_F_ and **E**_vb,InP_ from the InP band gap (**E**_g,InP_) (eq 7).

$$\begin{array}{c} E_{\mathrm{cb},s,\mathrm{InP}}=E_{g,\mathrm{InP}}-(E_{F}-E_{\mathrm{vb},\mathrm{InP}})-E_{\mathrm{bb}} \end{array}$$ 7

where **E**_g,InP_ = 1.35 eV and (**E**_F_ – **E**_vb,InP_) = 0.08 eV.

The energy of the conduction band minimum of each protection layer candidate (**E**_cb,PL_) referenced to **E**_F_ was calculated from eq 8.

$$\begin{array}{c} E_{\mathrm{cb},\mathrm{PL}}=E_{g,\mathrm{PL}}-(E_{F}-E_{\mathrm{vb},\mathrm{PL}}) \end{array}$$ 8

where **E**_g,PL_ is the band gap of the protection layer as measured by Tauc plot analysis and (**E**_F_ – **E**_vb,PL_) is the difference between the Fermi level and the valence band maximum of the protection layer, as determined by UPS.

The electron affinity for each protection layer (EA_PL_) was calculated from eq 9.

$$\begin{array}{c} {\mathrm{EA}}_{\mathrm{PL}}=\phi_{\mathrm{PL}}-E_{\mathrm{cb},\mathrm{PL}} \end{array}$$ 9

**E**_cb,s,InP_ and **E**_cb,PL_ were both referenced to the Fermi level. Consequently, the electronic alignment or misalignment (**E**_offset_) of InP and each protection layer was assessed via eq 10.

$$\begin{array}{c} E_{\mathrm{offset}}=E_{\mathrm{cb},\mathrm{PL}}-E_{\mathrm{cb},s,\mathrm{InP}} \end{array}$$ 10

A negative value for **E**_offset_ indicates that the conduction band minimum of the protection layer is more negative in energy than an electron coming from the p-InP, with the interfacial energetics being thermodynamically favorable for conduction of charge. Conversely, a positive value for **E**_offset_ indicates a thermodynamic barrier to interfacial charge conduction.

In prior studies, band diagrams derived from the energetics of n-Si | TiO_2_ interfaces have included the energetic potential drop (**E**_PD_) to account for the energy drop incurred as an electron travels across the resistive interfacial oxide.^7^ The native InP oxide is conductive, whereas silicon oxides are insulating,^16,17^ so **E**_PD_ was not included explicitly in the band diagrams determined herein for p-InP | oxide interfaces, but instead the entire interfacial energetic potential drop is contained in the quoted value of the interfacial dipole at the p-InP | oxide interface.

## Results

Angle-resolved XPS (ARXPS) measurements indicated that the native oxide on p-InP was indium-rich at the oxide | air interface, consistent with prior reports.^18–22^ The oxide also contained various intermixed oxide species, including InPO_4_ and In­(OH)_3_. This native oxide layer is collectively denoted herein as InPO_*x*_, with additional details on the composition of this oxide provided in Section S6.

Figure 1e shows the band diagram for etched p-InP that was coated with a native oxide surface layer. To construct this band diagram, the bulk energy levels were determined from literature values of 16.65 eV for the energy difference between the In 4d_5/2_ core-level and **E**_vb,InP_,^23^ 1.35 eV for **E**_g,InP_,^24^ and 4.38 eV for EA_InP_.^15^ The Fermi level position was calculated as described in the Experimental Methods section, and was 0.08 eV above **E**_vb,InP_. All other bulk InP values were calculated from these values.

**Figure 1:**
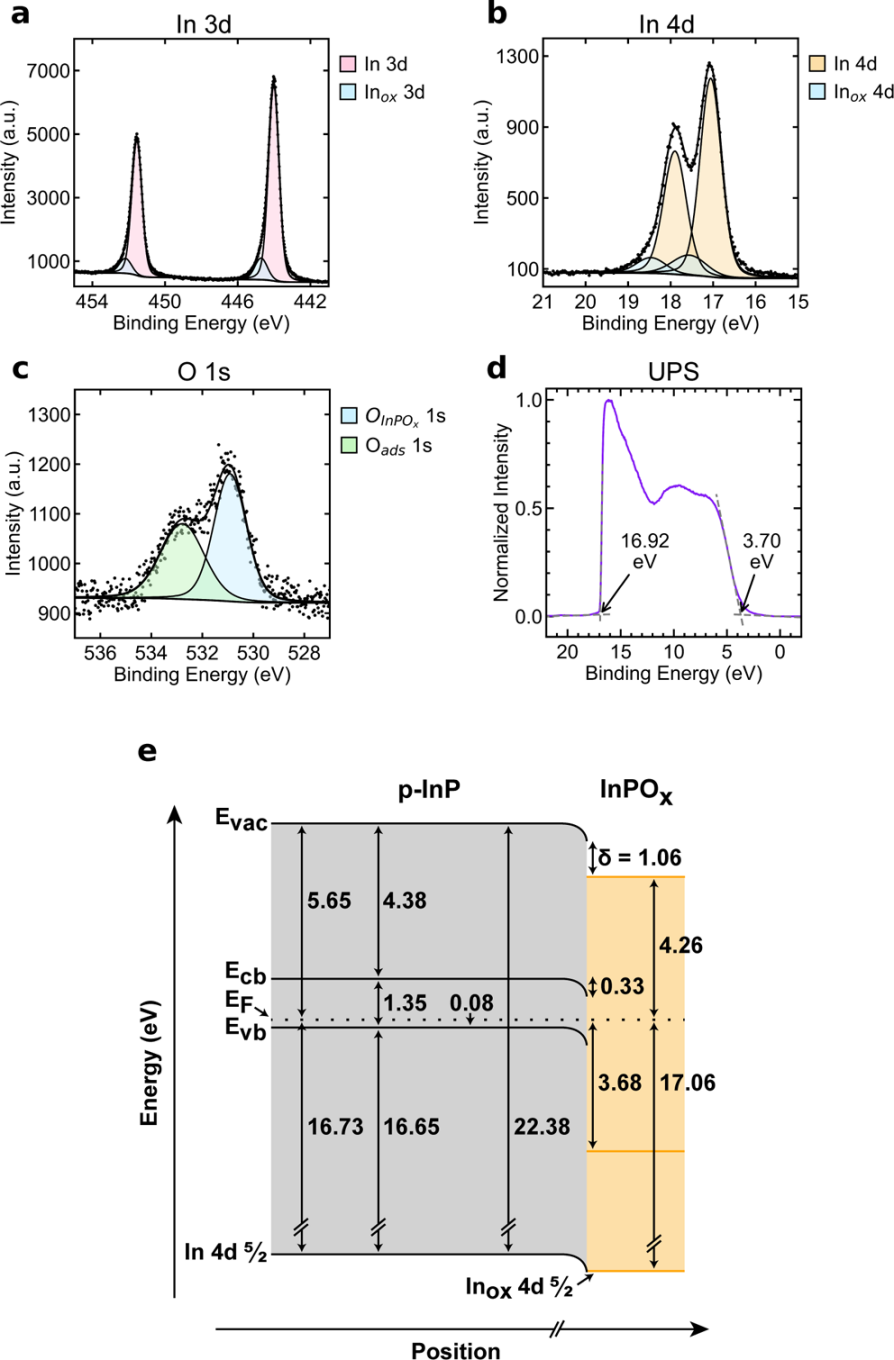
XPS data of the In 3d emissions (a), the In 4d emissions (b), and the O 1s emissions (c) from a representative sample of etched p-InP. O_ads_ denotes oxygen originating from adsorbed species on the sample surface. (d) UPS data of etched p-InP. The initial rise and the secondary electron cutoff are indicated with arrows. (e) Band diagram of etched p-InP. Energy values labeled in (d) correspond to a single representative acquisition and differ slightly from the average UPS-derived values in (e).

UPS and XPS measurements were used to determine the energy levels of the surface InPO_*x*_ layer. As stated in the Experimental Methods, five spectra for each energy region were collected from different locations on the sample. The values reported in Figure 1e represent the average value for each energy region from each of the five acquisitions. These average values may deviate slightly from the selected spectra shown in Figure 1a–d. Accordingly, the energy values explicitly labeled in the UPS data (Figure 1d), which correspond to a single representative acquisition, do not exactly match the average UPS-derived values reported in Figure 1e. All spectra for each energy region of each sample are available in the Supporting Information, Section S4. The In 4d_5/2_ core-level binding energy, measured by XPS, was 17.06 ± 0.101 eV, yielding (eq 4) a band bending (**E**_bb_) of 0.33 ± 0.101 eV. As determined by UPS, the energy difference between **E**_vb,InPO_*x*__ and **E**_F_ was 3.68 ± 0.105 eV. The secondary electron cutoff (SECO) in the UPS spectra was 16.94 ± 0.102 eV, meaning the work function of the surface InPO_*x*_ layer was 4.26 ± 0.102 eV (eq 5). Using the work functions of bulk InP and InPO_*x*_, along with **E**_bb_, the interfacial dipole (δ) was calculated (eq 6) as 1.06 ± 0.144 eV.

Figure 2 presents the band diagram for p-InP coated with a TiO_2_ protection layer. The energy levels of TiO_2_ were determined through a combination of XPS, UPS, and optical measurements, enabling calculation of the band alignment at the interface. The addition of an overlayer, such as TiO_2_, could change **E**_bb_ and δ, so these values were calculated independently for the InP | InPO_*x*_ | TiO_2_ system as well as for all other metal oxide overlayers. All values for bulk p-InP from Figure 1 were used without correction.

**Figure 2:**
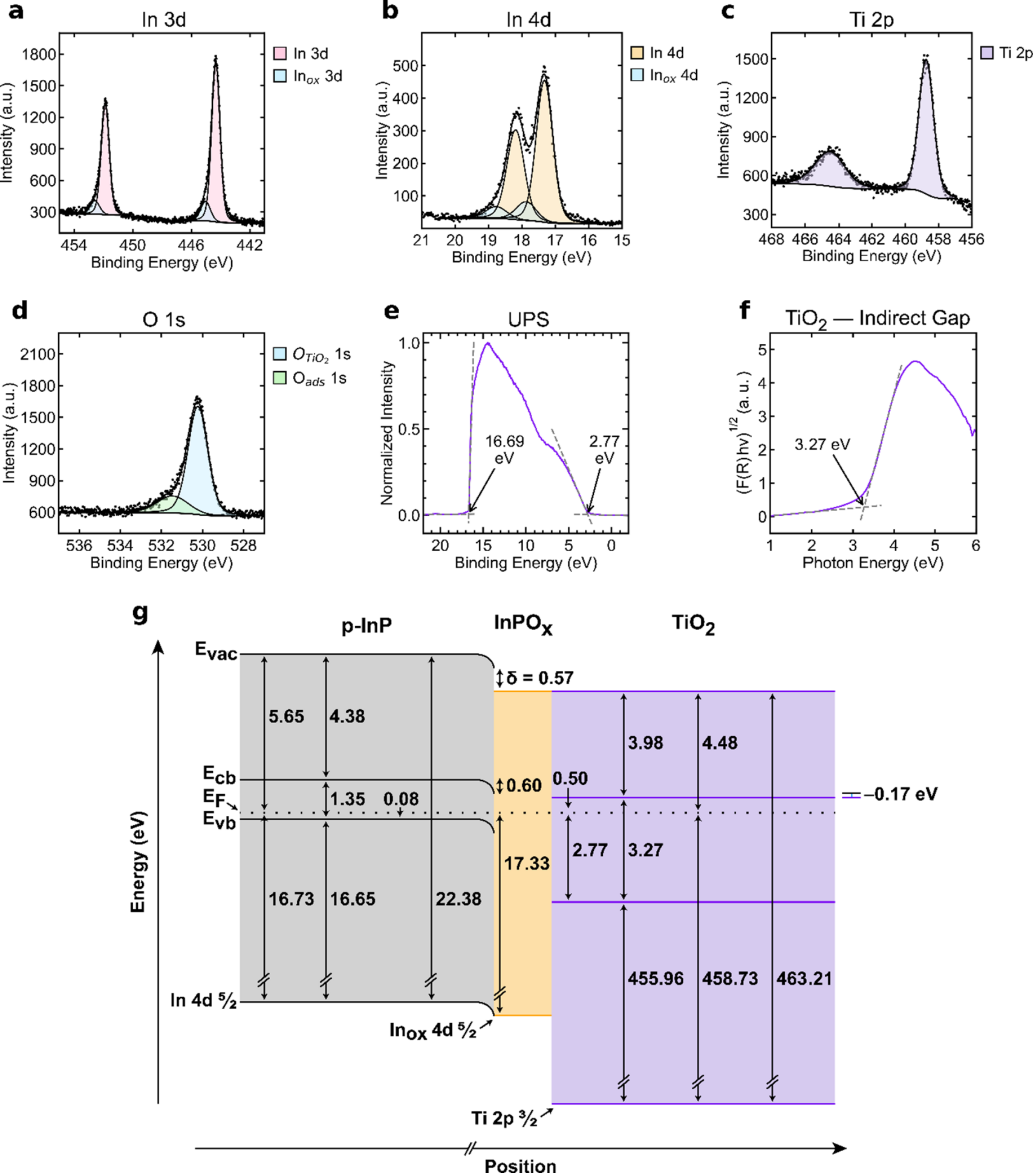
XPS data for the In 3d emissions (a), In 4d emissions (b), Ti 2p emissions (c), and the O 1s emissions (d) in a representative TiO_2_-coated p-InP sample. O_ads_ denotes oxygen originating from adsorbed species on the sample surface. (e) UPS data for the TiO_2_-coated p-InP sample. The initial rise and the secondary electron cutoff are indicated with arrows. (f) Tauc plot of TiO_2_ deposited on quartz. The band gap is indicated with an arrow. (g) Band diagram of the TiO_2_-coated p-InP system. Energy values labeled in (e) correspond to a single representative acquisition and differ slightly from the average UPS-derived values in (g).

For the TiO_2_ layer, XPS measurements indicated a binding energy of 458.73 ± 0.100 eV from the Ti 2p_3/2_ core-level relative to **E**_F_. This value, together with the corresponding O 1s peak position of 530.23 ± 0.100 eV, is consistent with values reported previously for stoichiometric TiO_2_, confirming the expected oxidation state of the deposited film.^25–27^ No evidence for a defect band arising from Ti­(III) species was obtained from UPS data, providing further evidence of the stoichiometric TiO_2_ composition. UPS measurements further indicated that the difference between **E**_vb,TiO_2__ and **E**_F_ was 2.77 ± 0.101 eV, and the average SECO was 16.72 ± 0.103 eV, meaning the average work function of the TiO_2_ layer was 4.48 ± 0.103 eV (eq 5). Tauc plot analysis indicated a band gap for TiO_2_ of **E**_g,TiO_2__ = 3.27 ± 0.100 eV, in agreement with prior results.^6^ The remaining interfacial energetic values were calculated using the results of these measurements. The energy difference from the Fermi level to **E**_cb,TiO_2__ was calculated to be 0.50 ± 0.142 eV (eq 8), and the electron affinity for TiO_2_ (EA_TiO_2__) was calculated to be 3.98 ± 0.176 eV (eq 9).

The core-level binding energy of the In 4d_5/2_ peak, measured via XPS, was 17.33 ± 0.100 eV, yielding (eq 4) a band bending (**E**_bb_) of 0.60 ± 0.100 eV. The interfacial dipole (δ) was calculated (eq 6) as 0.57 ± 0.144 eV. In combination with previously determined energy levels of InP (Figure 1), these values allowed construction of an energy band diagram that describes the heterojunction between p-InP and TiO_2_. From eq 7, the energy of an electron at the p-InP | metal oxide interface (**E**_cb,s,InP_) was calculated as 0.67 ± 0.100 eV more positive than **E**_F_. **E**_cb,TiO_2__ was 0.50 ± 0.142 eV more positive than **E**_F_ (eq 8), which means that **E**_offset_ was −0.17 ± 0.174 eV (eq 10) indicating a thermodynamically favorable electron flow (within uncertainty) from p-InP to the conduction band of TiO_2_.

Figure 3 displays the band diagram for p-InP coated with a Nb_2_O_5_ protection layer. As with the TiO_2_-coated p-InP system, to establish the band alignment at the interface, the energy levels of Nb_2_O_5_ were determined using a combination of XPS, UPS, and optical absorption measurements. XPS measurements indicated a binding energy for the Nb 3d_5/2_ core-level of 207.29 ± 0.101 eV relative to **E**_F_ and a binding energy of 530.72 ± 0.107 eV for the O 1s electrons, consistent with stoichiometric Nb_2_O_5_.^28,29^ No defect band attributable to Nb suboxides was observable in the UPS data. The energy difference between **E**_vb,Nb_2_O_5__ and **E**_F_ was 2.86 ± 0.107 eV, and the work function of the Nb_2_O_5_ layer was 4.53 ± 0.102 eV (eq 5). From Tauc plot analysis, the band gap of Nb_2_O_5_ was measured as **E**_g,Nb_2_O_5__ = 3.53 ± 0.100 eV. From these values, the energy difference between **E**_F_ and **E**_cb,Nb_2_O_5__ was determined to be 0.67 ± 0.146 eV (eq 8), and EA_Nb_2_O_5__ was calculated as 3.86 ± 0.178 eV (eq 9).

**Figure 3:**
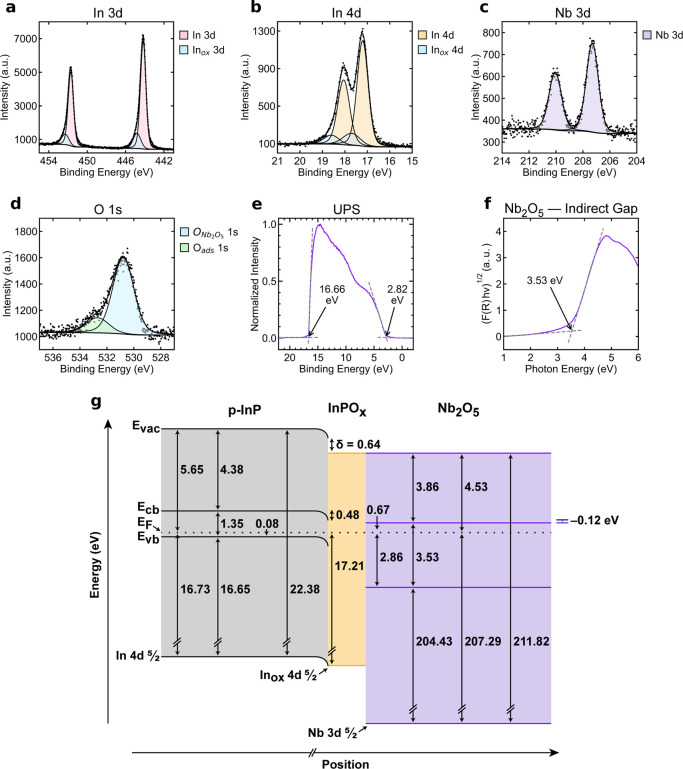
XPS data of the In 3d emissions (a), and the In 4d emissions (b), the Nb 3d emissions (c), and the O 1s peaks (d) in a representative Nb_2_O_5_-coated p-InP sample. O_ads_ denotes oxygen originating from adsorbed species on the sample surface. (e) UPS data of the Nb_2_O_5_-coated p-InP sample. The initial rise and the secondary electron cutoff are indicated with arrows. (f) Tauc plot of Nb_2_O_5_-deposited on quartz. The band gap is indicated with an arrow. (g) Band diagram of the Nb_2_O_5_-coated p-InP system. Energy values labeled in (e) correspond to a single representative acquisition and differ slightly from the average UPS-derived values in (g).

The core-level binding energy of the In 4d_5/2_ peak, measured via XPS, was 17.21 ± 0.101 eV, yielding (eq 4) a band bending (**E**_bb_) of 0.48 ± 0.101 eV. The interfacial dipole (δ) was calculated as 0.64 ± 0.144 eV, using eq 6. Figure 3g shows the energy band diagram for the p-InP | Nb_2_O_5_ heterojunction. The energy of an electron at the p-InP | Nb_2_O_5_ interface (**E**_cb,s,InP_) was calculated to be 0.79 ± 0.101 eV more positive in energy than **E**_F_ (eq 7). **E**_cb,Nb_2_O_5__ was (eq 8) 0.67 ± 0.146 eV more positive in energy than **E**_F_, indicating that **E**_offset_ was −0.12 ± 0.178 eV (eq 10). Like TiO_2_, Nb_2_O_5_ provides a thermodynamically favorable conduction pathway (within uncertainty) for interfacial electron transfer across the p-InP | metal oxide interface.

Figure 4 presents the band diagram for p-InP coated with a Ta_2_O_5_ protection layer. XPS measurements indicated a binding energy of 26.49 ± 0.101 eV for the Ta 4f_7/2_ core-level and a binding energy of 530.72 ± 0.101 eV for O 1s relative to **E**_F_, in accord with expectations for stoichiometric Ta_2_O_5_.^30,31^ No defect band was evident in the UPS data. The energy difference between **E**_vb,Ta_2_O_5__ and **E**_F_ was measured as 2.59 ± 0.103 eV, and the work function of Ta_2_O_5_ was measured as 4.67 ± 0.101 eV (eq 5). The band gap of Ta_2_O_5_ was measured as **E**_g,Ta_2_O_5__ = 4.23 ± 0.100 eV. Using these values, the energy difference between **E**_F_ and **E**_cb,Ta_2_O_5__ was calculated as 1.64 ± 0.144 eV (eq 8) and EA_Ta_2_O_5__ was determined to be 3.03 ± 0.176 eV (eq 9).

**Figure 4:**
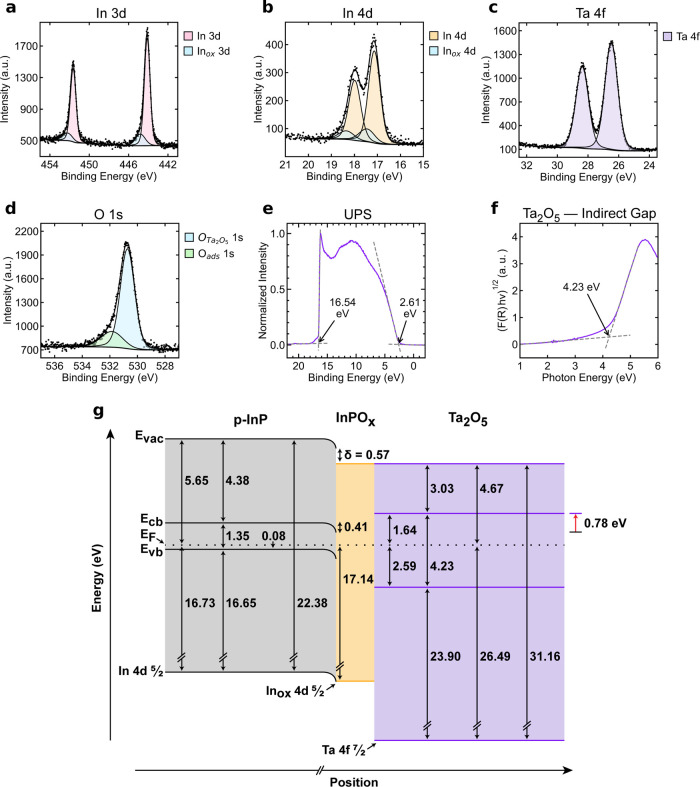
XPS data of the In 3d emissions (a), and In 4d emissions (b), the Ta 4f emissions (c), and the O 1s emissions (d) in a representative Ta_2_O_5_-coated p-InP sample. O_ads_ denotes oxygen originating from adsorbed species on the sample surface. (e) UPS data of the Ta_2_O_5_-coated p-InP sample. The initial rise and the secondary electron cutoff are indicated with arrows. (f) Tauc plot of Ta_2_O_5_-deposited on quartz. The band gap is indicated with an arrow. (g) Band diagram of the Ta_2_O_5_-coated p-InP system. Energy values labeled in (e) correspond to a single representative acquisition and differ slightly from the average UPS-derived values in (g).

The core-level binding energy of the In 4d_5/2_ peak, measured via XPS, was 17.14 ± 0.100 eV, yielding **E**_bb_ = 0.41 ± 0.100 eV via eq 4. The value of δ was calculated (eq 6) as 0.57 ± 0.142 eV. From eq 7, the energy of an electron at the p-InP | Ta_2_O_5_ (**E**_cb,s,InP_) interface was calculated as 0.86 ± 0.100 eV above **E**_F_. **E**_cb,Ta_2_O_5__ was (eq 8) 1.64 ± 0.144 eV above **E**_F_, meaning that **E**_offset_ was 0.78 ± 0.175 eV (eq 10). This thermodynamically unfavorable band alignment indicates that Ta_2_O_5_ should produce an insulating barrier rather than facilitating transfer of photogenerated minority carriers in p-InP across the InP | protection layer interface.

Figure 5 presents the completed band diagram for p-InP coated with a HfO_2_ protection layer. XPS measurements indicated a binding energy of 213.26 ± 0.103 eV for the Hf 4d_5/2_ core-level and 530.23 ± 0.102 eV for the O 1s level relative to **E**_F_, consistent with expectations for Hf­(IV) in HfO_2_.^32,33^ UPS measurements indicated that the energy difference between **E**_vb,HfO_2__ and **E**_F_ was 2.77 ± 0.120 eV, and the work function of HfO_2_ was determined to be 4.72 ± 0.105 eV (eq 5). From Tauc plot analysis, the band gap of HfO_2_ was measured as **E**_g,HfO_2__ = 5.61 ± 0.100 eV. The energy difference between **E**_F_ and **E**_cb,HfO_2__ was calculated as 2.84 ± 0.156 eV (eq 8) and EA_HfO_2__ was calculated as 1.88 ± 0.188 eV (eq 9).

**Figure 5:**
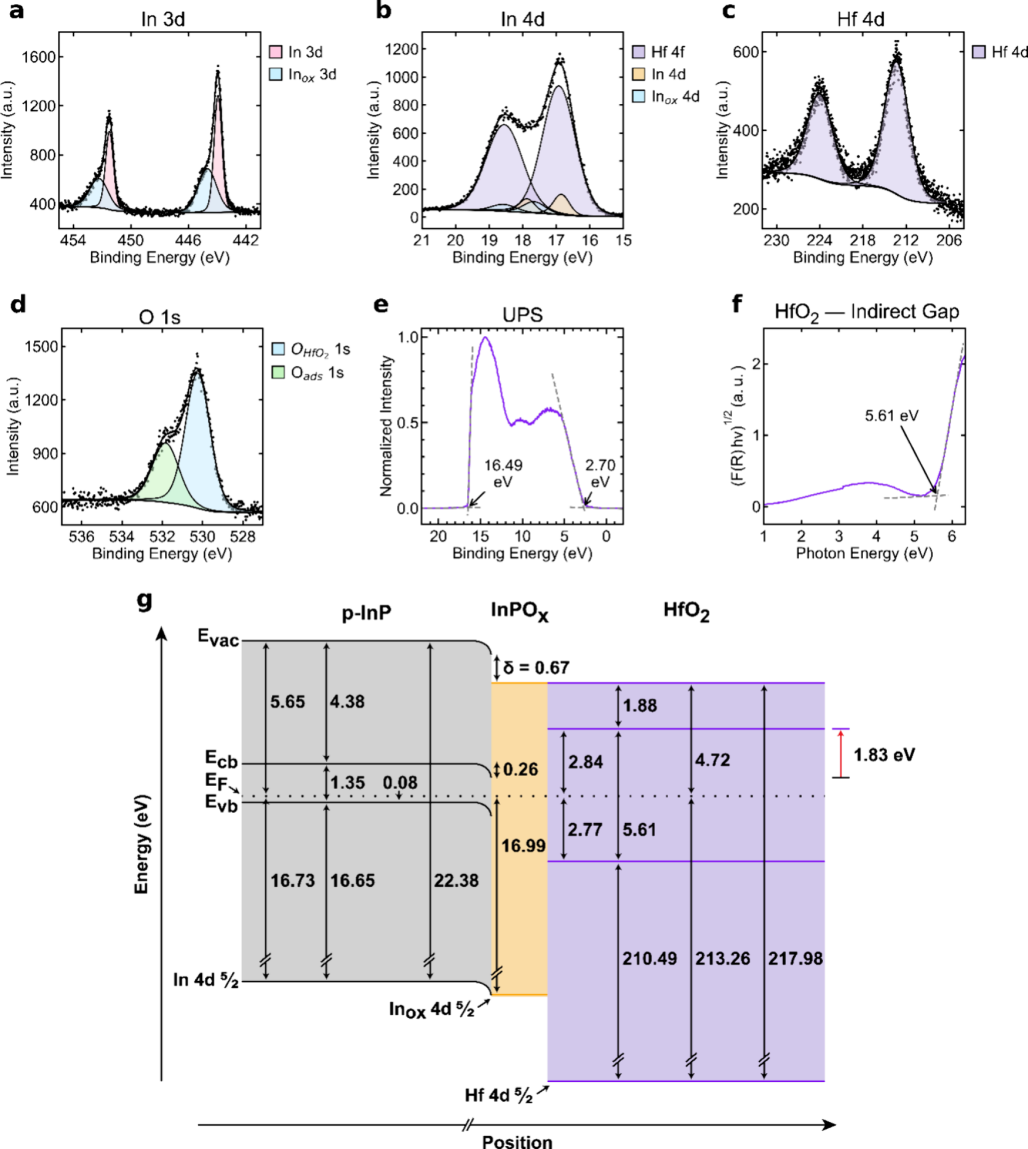
XPS data of the In 3d emissions (a), the In 4d emissions (b), the Hf 4d emissions (c), and the O 1s emissions (d) in a representative HfO_2_-coated p-InP sample. O_ads_ denotes oxygen originating from adsorbed species on the sample surface. (e) UPS data of the HfO_2_-coated p-InP sample. The initial rise and the secondary electron cutoff are indicated with arrows. (f) Tauc plot of HfO_2_-deposited on quartz. The band gap is indicated with an arrow. (g) Band diagram for the HfO_2_-coated p-InP system. Energy values labeled in (e) correspond to a single representative acquisition and differ slightly from the average UPS-derived values in (g).

The core-level binding energy of the In 4d_5/2_ peak was 16.99 ± 0.108 eV. As described in the Supporting Information, Section S7, the binding energy of the In 4d_5/2_ peak was deconvoluted from the overshadowing Hf 4f_7/2_ peak. From eq 4, this procedure yielded a value for **E**_bb_ of 0.26 ± 0.108 eV. The value of δ was calculated as 0.67 ± 0.151 eV (eq 6).

The energy of an electron at the p-InP | HfO_2_ interface (**E**_cb,s,InP_) was 1.01 ± 0.108 eV more positive than **E**_F_ (eq 7). **E**_cb,HfO_2__ was calculated to be 2.84 ± 0.156 eV above **E**_F_ (eq 8), meaning that **E**_offset_ was 1.83 ± 0.190 eV (eq 10). This conduction band misalignment should thermodynamically preclude electron injection, indicating that HfO_2_ should produce a barrier to interfacial electron transfer rather than a conductive protection layer for photogenerated minority carriers in p-InP.

Table 2 summarizes all of the numerical values explicitly referenced in the text related to Figures 1–5.

**Table 2:** Summary of Text-Referenced Numerical Values for Figures 1–5 ^a^

	Etched	TiO_2_-coated	Nb_2_O_5_-coated	Ta_2_O_5_-coated	HfO_2_-coated
**E**_vb,InP_ – bulk In 4d_5/2_	16.65	16.65	16.65	16.65	16.65
**E**_g,InP_	1.35	1.35	1.35	1.35	1.35
EA_InP_	4.38	4.38	4.38	4.38	4.38
**E**_F_ – **E**_vb,InP_	0.08	0.08	0.08	0.08	0.08
**E**_F_ – PL core level atom		458.73 (Ti 2p_3/2_)	207.29 (Nb 3d_5/2_)	26.49 (Ta 4f_7/2_)	213.26 (Hf 4d_5/2_)
**E**_F_ – **E**_vb,PL_	3.68	2.77	2.86	2.59	2.77
ϕ_PL_	4.26	4.48	4.53	4.67	4.72
**E**_g,PL_		3.27	3.53	4.23	5.61
**E**_cb,PL_ – **E**_F_		0.50	0.67	1.64	2.84
EA_PL_		3.98	3.86	3.03	1.88
**E**_bb_	0.33	0.60	0.48	0.41	0.26
δ	1.06	0.57	0.64	0.57	0.67
**E**_cb,s,InP_		0.67	0.79	0.86	1.01
**E**_offset_		–0.17	–0.12	0.78	1.83

a All values are in eV.

## Discussion

### Electrode Composition

The electrodes studied herein consisted of p-type InP with a thin indium-rich oxide (InPO_*x*_) that formed even under brief air exposure, followed by an ALD-grown protection overlayer (TiO_2_, Nb_2_O_5_, Ta_2_O_5_, or HfO_2_). Angle-resolved XPS indicated that the native interfacial oxide was compositionally graded and enriched in In toward the outermost surface, with contributions from InPO_4_ and In­(OH)_3_, consistent with expectations for etched InP surfaces.^18–22^ Within this common interfacial structure, the ALD films were stoichiometric. The Ti 2p_3/2_ (∼458.7 eV) and O 1s (∼530.2 eV) for TiO_2_; Nb 3d_5/2_ (∼207.3 eV) and O 1s (∼530.7 eV) for Nb_2_O_5_; Ta 4f_7/2_ (∼26.5 eV) and O 1s (∼530.7 eV) for Ta_2_O_5_; and Hf 4d_5/2_ (∼213.3 eV) and O 1s (∼530.2 eV) for HfO_2_ XPS values were in accord with expectations for fully oxidized cations in the oxide protection layer. Moreover, no suboxide defect bands were detectable by UPS. Tauc analyses on identically prepared films deposited on quartz yielded optical gaps in the expected ranges for each oxide. The functional heterojunctions are thus best described as p-InP | InPO_*x*_ | metal oxide, where the metal oxides are stoichiometric, wide band gap oxides.

### Photoelectron Measurements

TiO_2_ and Nb_2_O_5_ had conduction band minima that were more negative in energy (within uncertainty) than the energy of an electron at the p-InP | metal oxide interface. This alignment provides a thermodynamically favorable pathway for electron extraction into the oxide, consistent with cathodic conduction from p-InP through these oxides.^11^ The values of **E**_offset_ for TiO_2_ and Nb_2_O_5_ were negative within uncertainty, meaning that a small thermodynamic barrier may exist. Typically, a 59 meV increase in barrier height at 298 K corresponds to an order-of-magnitude decrease in an ideal activated rate of interfacial charge transfer. In operation of a photocathode, the small barriers possibly imposed by TiO_2_ and Nb_2_O_5_ could manifest as modest changes in the photocurrent onset or fill factor rather than complete suppression of the photocurrent, especially if defect- or state-mediated transport pathways are available.

In contrast, Ta_2_O_5_ and HfO_2_ had conduction band minima that were more positive in energy than **E**_cb,s,InP_ producing a large energetic barrier that blocks electron transport, consistent with prior findings.^11^ All four oxides had valence band maxima that were substantially more negative in energy than **E**_vb,s,InP_. This offset in valence band maxima blocks hole transport to the electrolyte through the protection layer, explaining the absence of anodic current in p-InP electrodes coated with any of the metal oxides studied here.^11^ Blocking hole transport prevents dark anodic corrosion at open circuit, inhibiting oxidative degradation of the electrode.^11^ Table 2 provides a consolidated summary of the energetic quantities used in this analysis.

### Energetics of Oxide Protection Layers

A further consideration is whether sample-to-sample variations in the measured adventitious C 1s binding energy, before referencing to 285.00 eV, could reflect a surface dipole that shifts the vacuum level and is not explicitly accounted for in the UPS data, because UPS data do not have a universal reference for the vacuum level. Prior to referencing, the average C 1s binding energies were 285.36 eV for etched InP, 284.89 eV for TiO_2_-coated InP, 285.30 eV for Nb_2_O_5_-coated InP, 285.42 eV for Ta_2_O_5_-coated InP, and 285.35 eV for HfO_2_-coated InP. Table S3 provides a complete listing of the individual C 1s values, along with their averages and standard deviations.

In a conservative estimate, if the full deviation of the measured C 1s peak from 285.00 eV was assumed to originate directly from a vacuum-level shift, then the work function and difference between **E**_F_ and **E**_vb_ calculated from UPS could be shifted differently between samples, also affecting **E**_offset_ for each sample. Applying this upper-bound assumption, **E**_offset_ would shift from −0.17 to −0.28 eV for TiO_2_, but would shift from −0.12 to 0.18 eV for Nb_2_O_5_, from 0.78 to 1.20 eV for Ta_2_O_5_, and from 1.83 to 2.18 eV for HfO_2_. We emphasize that this represents the maximum possible impact and is unlikely to apply in full, because deviations in the peak of the adventitious C 1s emission can arise from multiple contributions that do not correspond to a true surface vacuum-level shift in the UPS data, such as charging during the XPS measurements.

In contrast to systems with insulating native oxides, such as Si | SiO_2_, for which an explicit interfacial energetic potential drop must be included in the construction of band diagrams, the native oxide remaining on etched InP is sufficiently conductive that no additional potential drop was modeled in this study.^16,17^ In nondegenerately doped Si | SiO_2_ systems (with or without TiO_2_), the reported potential drop ascribed to the insulating oxide was on the order of 0–0.15 eV.^7^ For p-type systems, the potential drop decreases the energy of an electron from the semiconductor. A correction of this magnitude, if applied to the InP | InPO_*x*_ | metal oxide system, would place **E**_cb_ of TiO_2_ and Nb_2_O_5_ roughly equal to, or still slightly more negative than, the energy of an electron from InP. Larger potential drops, of 0.4–0.5 eV, have been reported for degenerately doped systems,^7^ but the InP samples in this study were not degenerately doped. A small potential drop that is similar across all samples would shift the absolute energy values uniformly without affecting the relative trends among the metal oxides used in this work.

When no defect bands are present, effective photocathode protection therefore requires meeting two energetic criteria at the actual InP | InPO_*x*_ | metal oxide interface. Specifically, for electron extraction via alignment of the conduction band edges: **E**_cb_ for the metal oxide must equal to or more negative than the energy of an electron at the surface of the illuminated p-InP. Practically, **E**_cb_/*q* should also be equal to or more negative than the RHE potential. Also, for hole blocking via misalignment of **E**_vb_: **E**_vb_ for the metal oxide must be more negative than **E**_vb,s,InP_ so that hole injection into the metal oxide is energetically unfavorable, suppressing oxidative degradation and recombination pathways. TiO_2_ and Nb_2_O_5_ simultaneously satisfy both conditions when used with p-InP electrodes, consistent with the observed electron conduction of these oxides while preventing degradation of the etched p-InP surface.^11^ Ta_2_O_5_ and HfO_2_ meet the hole blocking requirement but do not meet the electron extraction requirement, consistent with the behavior of these metal oxides as barriers to interfacial transfer of photogenerated minority carriers in p-InP photocathodes. The trends in conduction band positions for the various oxide protection layers are consistent with expectations for a relatively constant valence band position for the various oxides, reflecting the predominant O 2p character of the valence bands in these systems, with the valence band energies in all the oxides investigated herein located at ∼−7.2 eV from the local vacuum level. Consequently, increases in the band gap of the oxides (**E**_g,TiO_2__ < **E**_g,Nb_2_O_5__ < **E**_g,Ta_2_O_5__ < **E**_g,HfO_2__) resulted in more positive conduction band energies relative to the local vacuum level. Despite some variation in the magnitude of the interfacial dipole at the various p-InP | oxide interfaces, the change in the conduction band energy of the various oxides unfavorably increases the energetic barriers for transfer of photogenerated minority carriers from the p-InP into the oxide protection layer as the band gap of the oxide increases.

### Characteristics of Oxide Protection Layers

Although TiO_2_ and Nb_2_O_5_ are expected to facilitate injection of photogenerated minority-carrier electrons in p-InP under the conditions studied, the energy levels in metal oxide layers can be affected by deposition parameters and postdeposition treatments. ALD conditions such as temperature, the choice of oxidant (e.g., O_3_ vs H_2_O), and precursor pulse times can affect the stoichiometry, defect density, and electronic structure of the resulting oxide films.^34,35^ Annealing after deposition may shift the energy levels and/or modify the band gap of the oxide, and may also influence the interfacial trap density.^36,37^ In systems where **E**_cb_ of the oxide initially is more positive than the energy of photogenerated minority-carrier electrons in the semiconductor, modifications to the chemistry of the oxide or to the interfacial conditions may enable conduction of these charge carriers through the interface. Dipole engineering at the interface due to adsorbed species can shift the energy levels relative to the vacuum level.^38,39^ For instance, adsorption of sulfur has been used to passivate InP surfaces,^40–42^ and adsorption of chalcogenides shifts the band edges of II–VI photoanodes.^43–46^ Functionalization of Si with methyl groups shifts the band edges of Si photoelectrodes,^47–50^ and alkylation of InP may shift the band edges of InP photoelectrodes.^51^ Alternatively, the creation of midgap states or defect states may facilitate transport across oxide layers that otherwise would block interfacial electron transfer. For example, hole conduction in an n-Si | oxide | TiO_2_ system has been attributed to a TiO_2_ defect band positioned at the appropriate energy.^7^

The electrical and electrochemical properties of the oxide | catalyst | electrolyte interface are also important factors in determining the performance of catalyst-coated protection layers to effect fuel-forming electrochemical half-reactions. For example, TiO_2_ photoanode protection layers form low barrier, ohmic contacts to Ni oxy-hydroxide catalysts for the oxygen-evolution reaction in alkaline electrolytes, but carrier flow at this interface is impeded when metals with high work functions, such as Pt or Ir, are used in such systems as catalysts.^52,53^ Consequently, separate in situ investigations are required to understand the energetic and kinetic barriers at the oxide | catalyst | liquid interface in specific systems of interest.

### Extrapolation to GaInP

Gallium indium phosphide (GaInP) is a high-performance III–V semiconductor that has been implemented in tandem PEC architectures, in which both the photoanode and photocathode receive direct illumination.^54,55^ In contrast to side-by-side designs, tandem PECs can produce high current densities per unit area and large solar-to-hydrogen efficiencies by effectively utilizing the solar spectrum. GaInP is especially advantageous in such configurations due to its tunable band gap and favorable band edge positions. Because GaInP and InP exhibit nearly identical degradation mechanisms under PEC conditions,^56–58^ the findings presented in this work may also help guide the design of protection strategies for p-GaInP.

**E**_cb,GaInP_ is ∼0.5 to 1 eV more positive than **E**_cb,InP_, depending on the ratio of Ga to In.^59,60^ Consequently, protection layers that exhibit misalignment of the positions of their conduction band edges with InP due to relatively positive **E**_cb_ values, such as Ta_2_O_5_ (**E**_cb,Ta_2_O_5__ is 0.78 eV more positive than **E**_cb,s,InP_), may nonetheless form energetically favorable junctions with some compositions of p-GaInP, specifically alloys with a composition above the direct to indirect gap transition.^60,61^ The methodology described herein, including band alignment characterization via photoelectron spectroscopy, could thus be extended to screen candidate oxides for GaInP, enabling identification of protection layers that ensure both chemical stability and interfacial charge transport of photogenerated minority carriers through the protection layer.

## Conclusions

TiO_2_- and Nb_2_O_5_-coated p-InP photocathodes facilitate electron transfer, in contrast to Ta_2_O_5_- and HfO_2_-coated p-InP photocathodes. In this study, the conduction band alignment was identified as the key factor that determines the rate of interfacial electron transfer at each protected p-InP photocathode. Charge transfer readily occurred when **E**_cb_ of the protection layer was equal to or more negative in energy than the energy of an electron coming from illuminated p-InP. Protection layers with **E**_cb_ more positive than **E**_cb,s,InP_ formed barriers that inhibited electron conduction. Specifically, the TiO_2_ and Nb_2_O_5_ protection layers had **E**_cb_ more negative (within uncertainty) than **E**_cb,s,InP_ by 0.17 and 0.12 eV, respectively, whereas Ta_2_O_5_ and HfO_2_ protection layers had **E**_cb_ more positive than **E**_cb,s,InP_ by 0.78 and 1.83 eV, respectively. All studied oxides had valence band maxima that were substantially lower in energy (∼2.5 eV) than **E**_vb,s,InP_, preventing facile interfacial hole transfer and inhibiting oxidative degradation. Together, these findings establish two energetic criteria for protective overlayers on p-InP: a conduction band minimum energy no higher than the electron energy at the p-InP surface to permit electron extraction, and a sufficiently low energy valence band maximum to block holes and prevent oxidative degradation of p-InP photocathodes.

## Supplementary Material



## Notes

Instrument settings, peak fitting constraints, uncertainty calculations, carbon referencing, all XPS and UPS values used in this work, information on construction of band diagrams, surface characterization of InPO_x_, and deconvolution of Hf 4f and In 4d spectra (PDF)
