# Supplementary material for: Photoelectron Spectroscopic Determination of the Interfacial Energetics of Metal Oxide Protection Layers on p‑InP Photocathodes
Source: J Phys Chem C Nanomater Interfaces. 2026 Feb 23;130(9):3548–59. doi: 10.1021/acs.jpcc.5c08203 (PMC12969368; doi:10.1021/acs.jpcc.5c08203)
Supplement: Supplementary file 1 [file jp5c08203_si_001.pdf]

Supporting Information for "Photoelectron Spectroscopic Determination of the Interfacial Energetics of Metal Oxide Protection Layers on p-InP Photocathodes"

Authors: *Dominic Covelli*[1], *Alexandre Z. Ye* [1], *Jake M. Evans* [1], *Ty A. Schaller*[1], *Xinyi Elaine Shen*[1], *Paul J. L. Bean*[1], *Nathan S. Lewis* [1,2,*]

1. Division of Chemistry and Chemical Engineering, California Institute of Technology, Pasadena, CA 91125, United States
2. Beckman Institute, California Institute of Technology, Pasadena, CA 91125, United States

*. E-Mail: nslewis@caltech.edu. Tel: (626) 395-6335

Table of Contents:

1. Instrument Settings and Peak Fitting Constraints
2. Uncertainty Calculations
3. Measurement Referencing
4. All XPS and UPS Data
5. Construction of Band Diagrams
6. Surface Characterization of $InPO_x$
7. Deconvolution of Hf 4f and In 4d spectra
8. References

1. Instrument Settings and Peak Fitting Constraints

XPS/UPS Settings

Region	Center eV	Width eV	Pass Energy	Step eV	Dwell Time (ms)	Sweeps
**C 1s**	285	16	5	0.025	100	10
**O 1s**	532	18	5	0.025	100	10
**P 2p**	129	15	5	0.025	100	10
**In 3d**	447.95	20.1	5	0.025	100	10
**In 4d/ Hf 4f**	11	24	5	0.025	100	10
**Ti 2p**	462	18	5	0.025	100	10
**Nb 3d**	208	13.1	5	0.025	100	10
**Ta 4f**	27	14	5	0.025	100	10
**Hf 4d**	219	30	5	0.025	100	10
**Valence Band (UPS)**	10	24	5	0.05	100	5

Table S1: XPS/UPS settings used in the collection of data for this work. These settings were used for the collection of all spectra except for the angle-resolved XPS (ARXPS) data. The ARXPS settings are provided in Table S2.

ARXPS Settings

Region	Center eV	Width eV	Pass Energy	Step eV	Dwell Time (ms)	Sweeps
**C 1s**	285	16	20	0.025	100	20
**O 1s**	532	18	20	0.025	100	20
**P 2p**	129	15	20	0.025	100	20
**In 3d**	447.95	20.1	20	0.025	100	20
**In 4d/ Hf 4f**	11	24	20	0.025	100	20
**Ti 2p**	461	14	20	0.025	100	20
**Nb 3d**	208	13.1	20	0.025	100	20
**Ta 4f**	27	14	20	0.025	100	20
**Hf 4d**	219	30	20	0.025	100	20

Table S2: ARXPS settings used in the collection of data shown in Section S6.

For a given background, peaks were defined by their line shape, full width at half maximum (FWHM), position, and area. All XPS peaks were fit in CasaXPS using a mixed LA(50) line shape (50% Gaussian / 50% Lorentzian) with a Shirley background, except for the Hf 4f peaks, which were fit using the LA(5) line shape and a Shirley background. The default CasaXPS FWHM constraints were not changed, except for the HfO_2 on p-InP dataset, where

substantial overlap of the Hf 4f and In 4d envelopes required additional deconvolution (see Section S7). For all datasets, the only position with a single-value constraint (no range) was the In_{ox} $4d_{3/2}$ peak, which was fixed in position at 0.90 eV higher in binding energy than the In_{ox} $4d_{5/2}$ peak. This peak position is in accord with the spin-orbit separation observed between the In $4d_{5/2}$ and In $4d_{3/2}$ peaks in prior work.[1]

Here, 'In_{ox}' denotes the XPS component associated with oxidized indium in the native surface oxide, whereas 'In_{InP}' denotes the component corresponding to indium bound to phosphorus within the crystalline InP lattice. In the figures, this lattice component is labeled simply as 'In' for brevity.

For spin-orbit pairs, the area of the non-dominant component was constrained to the theoretical branching ratio relative to the dominant component (i.e. In_{InP} $4d_{3/2}$ area = 0.667 * In_{InP} $4d_{5/2}$ area).

2. Uncertainty Calculations

The following section describes the calculation of uncertainty (σ) values used in this work.

For XPS and UPS measurements, 5 measurements were recorded for each sample at different locations. The average value of these five measurements was used as the final value when constructing band diagrams. Measurement uncertainty was calculated using the standard error of the mean (SEM):

$$\sigma_{SEM} = \frac{Standard\ Deviation}{\sqrt{n}} \tag{S1}$$

Standard deviations were calculated using the sample formulation (dividing by n-1). The total uncertainty for a given measurement includes both the measurement uncertainty and the instrument uncertainty. For XPS and UPS measurements, the instrument uncertainty (σ_{inst}) was ± 0.10 eV.[2] The total uncertainty was therefore calculated as:

$$\sigma_{total} = \sqrt{\sigma_{SEM}^2 + \sigma_{inst}^2} \tag{S2}$$

The values used in the band diagrams are averages of the repeated measurements. Consequently, the plotted spectra may show a slightly different value than the value specified in the band diagram. In all figures, the spectrum whose measured value was closest to the mean was selected for display.

For each protection layer candidate, UV-Vis spectra were recorded from a single site on a quartz slide coated with 400 ALD cycles of that candidate. The optical band gaps, determined from Tauc plot extrapolations, were assigned an uncertainty of ±0.10 eV, which reflects the spectrophotometer’s wavelength accuracy, as well as the inherent variability in identifying the linear region of the Tauc plot.[3]

The uncertainty was propagated by addition in quadrature for any quantities calculated from two or more experimental values. As described in the Experimental Methods section, the dipole was calculated as:

$$\delta = \phi_{InP} - \left(\phi_{Overlayer} + \mathbf{E}_{bb}\right) \tag{6}$$

Both $\phi_{Overlayer}$ and $\mathbf{E}_{bb}$ have associated uncertainties, so the uncertainty in δ was calculated as:

$$\sigma_{\delta} = \sqrt{\sigma^2_{\phi_{Overlayer}} + \sigma^2_{\mathbf{E}_{bb}}} \tag{S3}$$

The uncertainty in the energy of an electron at the p-InP│metal oxide interface relative to the Fermi level ($\mathbf{E}_{cb,s,InP}$) (eq.7) was identical to the uncertainty in the band bending, because the band bending is the only term in eq. 7 with assigned uncertainty.

The conduction band energy of each protection layer ($\mathbf{E}_{cb,PL}$) referenced to $\mathbf{E}_F$ was calculated as:

$$\mathbf{E}_{cb,PL} = [\mathbf{E}_{g,PL} - (\mathbf{E}_F - \mathbf{E}_{vb,PL})] \tag{8}$$

The uncertainty in $\mathbf{E}_{cb,PL}$ was calculated as:

$$\sigma_{\mathbf{E}_{cb,PL}} = \sqrt{\sigma^2_{\mathbf{E}_{g,PL}} + \sigma^2_{(\mathbf{E}_F - \mathbf{E}_{vb,PL})}} \tag{S4}$$

The electron affinity for each protection layer (EA_{PL}) was calculated as:

$$EA_{PL} = \phi_{PL} - \mathbf{E}_{cb,PL} \tag{9}$$

The uncertainty in EA_{PL} was given by:

$$\sigma_{EA_{PL}} = \sqrt{\sigma^2_{\phi_{PL}} + \sigma^2_{\mathbf{E}_{cb,PL}}} \tag{S5}$$

The conduction band offset ($\mathbf{E}_{offset}$) was calculated as:

$$\mathbf{E}_{offset} = \mathbf{E}_{cb,PL} - \mathbf{E}_{cb,s,InP} \tag{10}$$

The corresponding uncertainty was calculated as:

$$\sigma_{\mathbf{E}_{offset}} = \sqrt{\sigma^2_{\mathbf{E}_{cb,PL}} + \sigma^2_{\mathbf{E}_{cb,s,InP}}} \tag{S6}$$

3. Measurement Referencing

In this work, XPS binding energies were aligned by referencing the adventitious C 1s peak to 285.00 eV, a standard procedure that corrects for rigid shifts caused by surface charging or static surface dipoles. This referencing process allows for accurate comparisons between XPS-derived quantities across different samples. However, the ultraviolet photoelectron spectroscopy (UPS) spectra were not corrected, because a common reference signal (such as C 1s) is not present in the UPS data. Consequently, combining UPS and XPS data, for example to determine interfacial dipoles, involves an implicit assumption that the vacuum-level offset between the UPS and XPS data is negligible and/or constant across samples. A full tabulation of the measured C 1s binding energies before referencing to 285.00 eV is provided in Table S3.

**Site**	**Etched**	**TiO_2-Coated**	**Nb_2O_5-Coated**	**Ta_2O_5-Coated**	**HfO_2-Coated**
**1**	285.27	284.87	285.24	285.40	285.39
**2**	285.36	284.89	285.28	285.43	285.37
**3**	285.40	284.87	285.32	285.35	285.31
**4**	285.34	284.90	285.33	285.48	285.40
**5**	285.42	284.93	285.32	285.44	285.28
**Average**	285.36	284.89	285.30	285.42	285.35
**Standard Deviation**	0.056	0.024	0.039	0.047	0.051

Table S3: Measured binding energy values (in eV) for C 1s peaks ascribable to adventitious C for each sample, before referencing to 285.00 eV. Values are reported to two decimal places, except for 'Standard Deviation' values, which are reported to three decimal places. Standard deviations were calculated using the sample formulation (dividing by n-1). The 'Average' and 'Standard Deviation' values were calculated before rounding.

4. All XPS and UPS Data

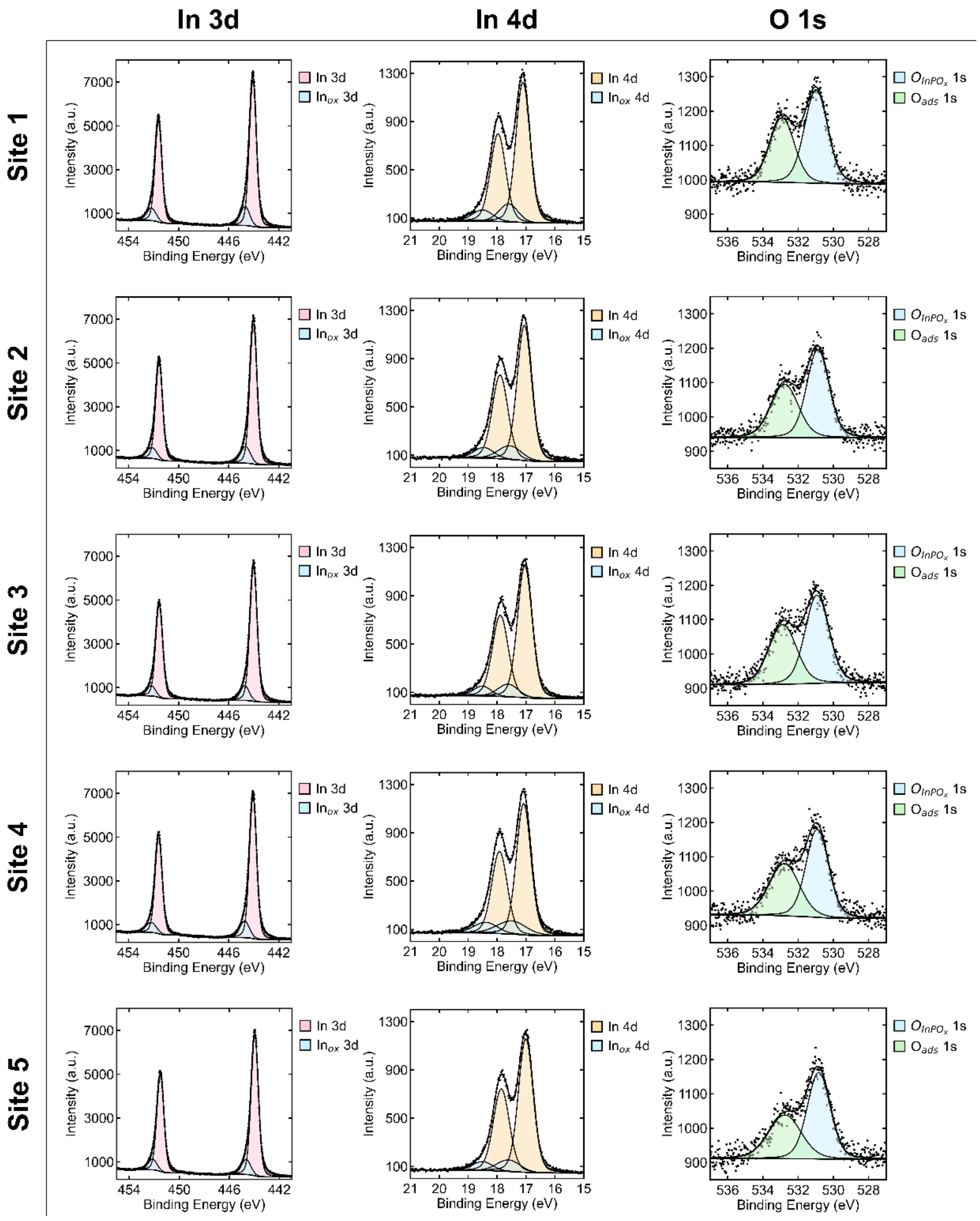

Figure S1: Replicate XP spectra from five separate locations on an etched p-InP photocathode.

Site	In_{InP} $3d_{5/2}$	In_{ox} $3d_{5/2}$	In_{InP} $4d_{5/2}$	In_{ox} $4d_{5/2}$	O 1s
**1**	444.07	444.70	17.11	17.61	530.98
**2**	444.02	444.66	17.06	17.56	530.88
**3**	444.02	444.71	17.04	17.62	530.92
**4**	444.06	444.62	17.08	17.50	530.91
**5**	443.97	444.66	17.01	17.61	530.82
**Average**	444.03	444.67	17.06	17.58	530.90
**Total Uncertainty**	0.101	0.101	0.101	0.102	0.103

Table S4: Binding energy values (in eV) for selected peaks from the spectra shown in Figure S1. Binding energy values were calibrated by correcting the carbon 1s emission ascribable to adventitious C to 285.00 eV and shifting all other binding energies by the same amount. Values are reported to two decimal places, except for 'Total Uncertainty' values, which are reported to three decimal places. The 'Average' and 'Total Uncertainty' values were calculated before rounding.

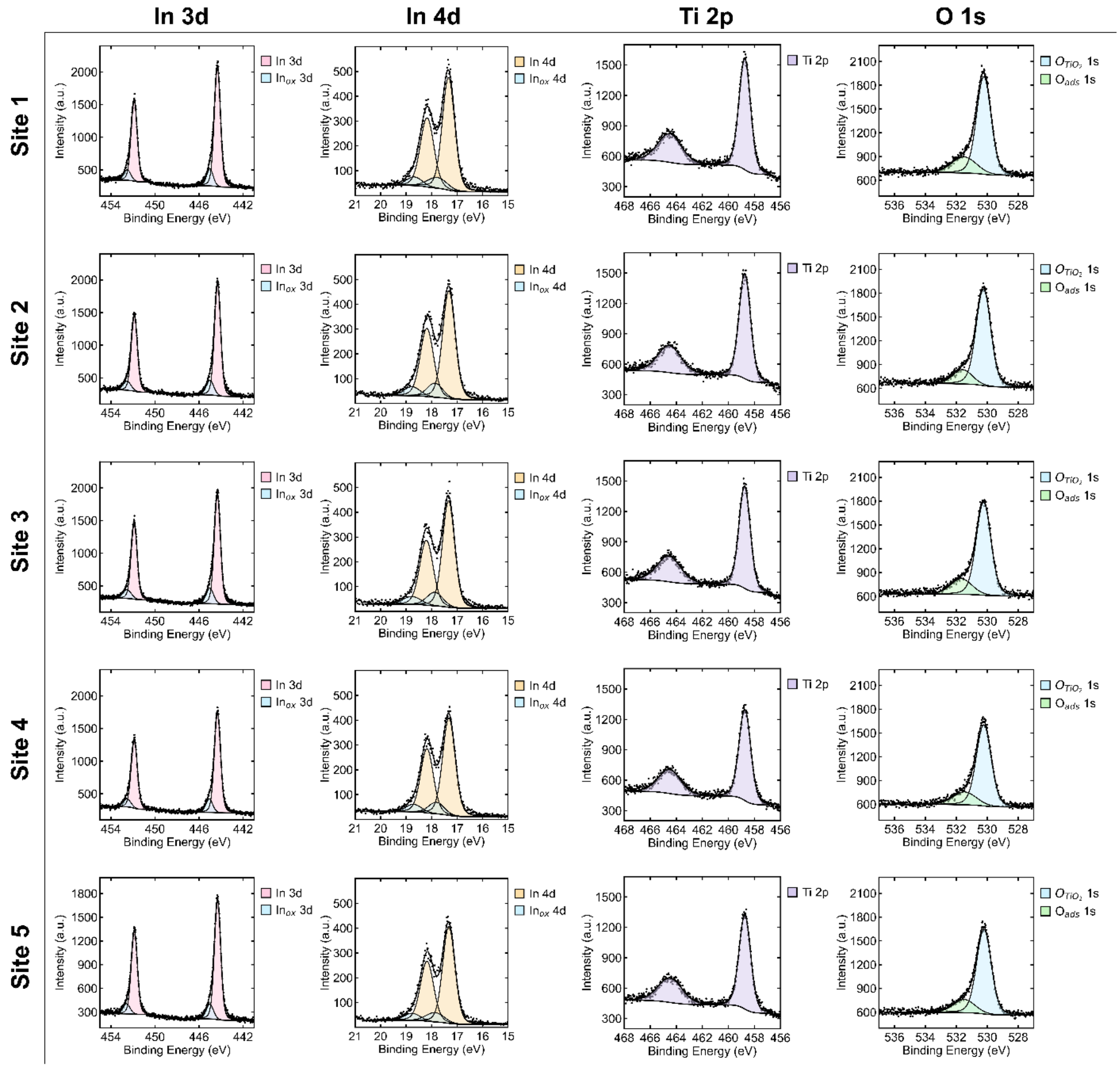

Figure S2: Replicate XP spectra from five separate locations on a TiO_2-coated p-InP photocathode.

Site	In_{InP} $3d_{5/2}$	In_{ox} $3d_{5/2}$	In_{InP} $4d_{5/2}$	In_{ox} $4d_{5/2}$	Ti $2p_{3/2}$	O 1s
**1**	444.34	445.02	17.33	17.78	458.74	530.22
**2**	444.32	445.01	17.32	17.87	458.74	530.24
**3**	444.34	445.02	17.33	17.87	458.75	530.23
**4**	444.33	445.08	17.32	17.81	458.73	530.22
**5**	444.32	445.03	17.32	17.86	458.73	530.21
**Average**	444.33	445.03	17.33	17.84	458.73	530.23
**Total Uncertainty**	0.100	0.101	0.100	0.102	0.100	0.100

Table S5: Binding energy values (in eV) for selected peaks from the spectra shown in Figure S2. Binding energy values were calibrated by correcting the carbon 1s emission ascribable to adventitious C to 285.00 eV and shifting all other binding energies by the same amount. Values are reported to two decimal places, except for 'Total Uncertainty' values, which are reported to three decimal places. The 'Average' and 'Total Uncertainty' values were calculated before rounding.

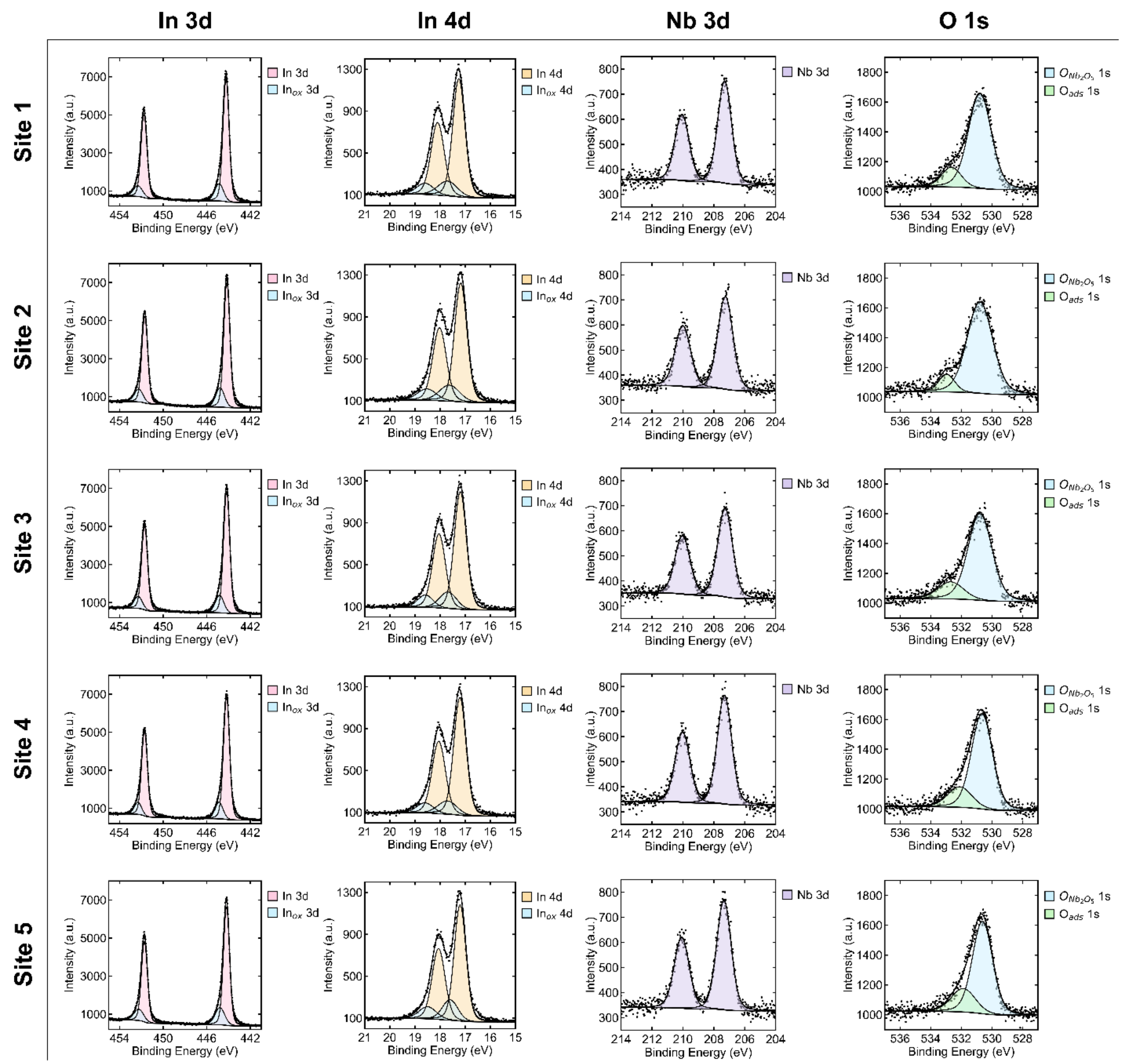

Figure S3: Replicate XP spectra from five separate sites on a Nb_2O_5-coated p-InP photocathode.

Site	In_{InP} $3d_{5/2}$	In_{ox} $3d_{5/2}$	In_{InP} $4d_{5/2}$	In_{ox} $4d_{5/2}$	Nb $3d_{5/2}$	O 1s
**1**	444.22	444.86	17.25	17.70	207.30	530.79
**2**	444.15	444.77	17.18	17.63	207.25	530.79
**3**	444.17	444.82	17.19	17.68	207.27	530.78
**4**	444.18	444.87	17.21	17.70	207.31	530.65
**5**	444.19	444.79	17.20	17.60	207.35	530.62
**Average**	444.18	444.82	17.21	17.66	207.29	530.72
**Total Uncertainty**	0.101	0.102	0.101	0.102	0.101	0.107

Table S6: Binding energy values (in eV) for selected peaks from the spectra shown in Figure S3. Binding energy values were calculated by correcting the carbon 1s emission ascribable to adventitious C to 285.00 eV and shifting all other binding energies by the same amount. Values are reported to two decimal places, except for ‘Total Uncertainty’ values, which are reported to three decimal places. The ‘Average’ and ‘Total Uncertainty’ values were calculated before rounding.

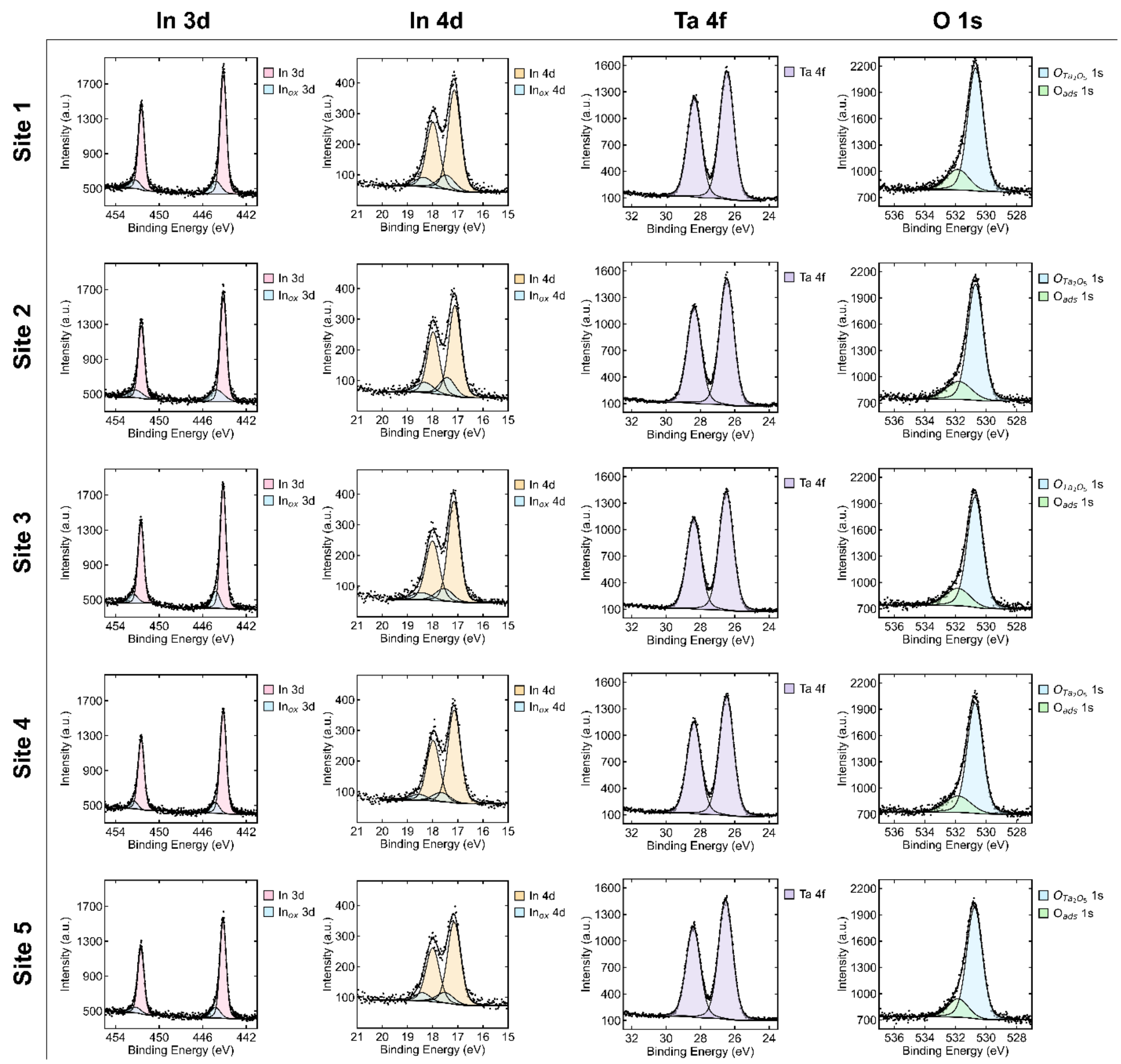

Figure S4: Replicate XP spectra from five separate locations on a Ta_2O_5-coated p-InP photocathode.

Site	In_{InP} $3d_{5/2}$	In_{ox} $3d_{5/2}$	In_{InP} $4d_{5/2}$	In_{ox} $4d_{5/2}$	Ta $4f_{7/2}$	O 1s
**1**	444.11	444.81	17.13	17.46	26.46	530.69
**2**	444.11	444.72	17.11	17.42	26.47	530.70
**3**	444.13	444.79	17.15	17.54	26.50	530.72
**4**	444.12	444.85	17.14	17.66	26.48	530.72
**5**	444.13	444.84	17.14	17.50	26.53	530.76
**Average**	444.12	444.80	17.14	17.51	26.49	530.72
**Total Uncertainty**	0.100	0.103	0.100	0.108	0.101	0.101

Table S7: Binding energy values (in eV) for selected peaks from the spectra shown in Figure S4. Binding energy values were calculated by correcting the carbon 1s emission ascribable to adventitious C to 285.00 eV and shifting all other binding energies by the same amount. Values are reported to two decimal places, except for ‘Total Uncertainty’ values, which are reported to three decimal places. The ‘Average’ and ‘Total Uncertainty’ values were calculated before rounding.

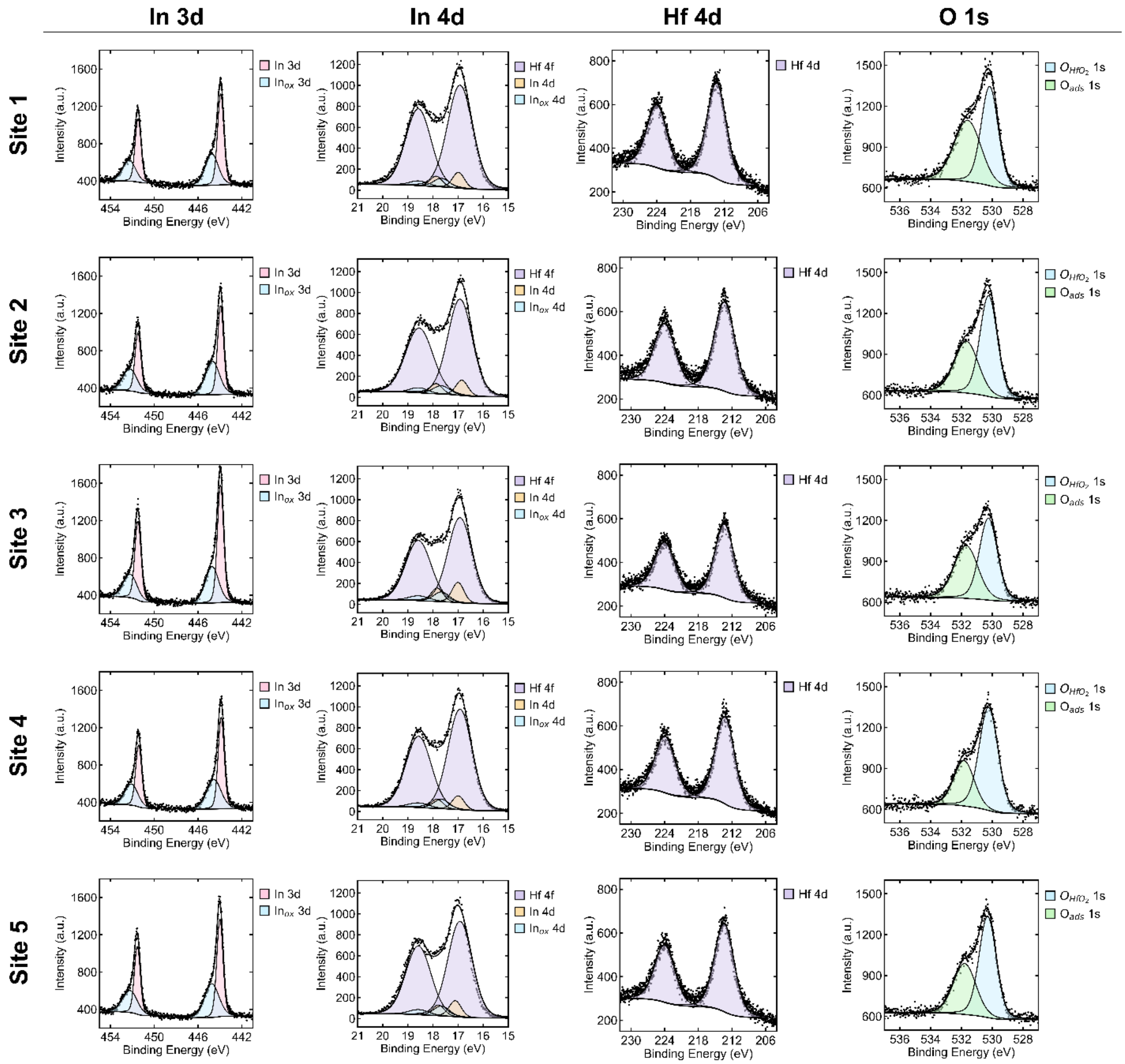

Figure S5: Replicate XP spectra from five separate locations on a HfO_2-coated p-InP photocathode.

Site	In_{InP} $3d_{5/2}$	In_{ox} $3d_{5/2}$	In_{InP} $4d_{5/2}$	In_{ox} $4d_{5/2}$	Hf $4d_{5/2}$	Hf $4f_{7/2}$	O 1s
**1**	443.94	444.75	16.98	17.66*	213.29	16.92*	530.16
**2**	443.94	444.69	16.99	17.66*	213.22	16.92*	530.23
**3**	443.96	444.73	17.00	17.66*	213.27	16.92*	530.25
**4**	443.89	444.55	16.85	17.66*	213.21	16.92*	530.23
**5**	444.01	444.75	17.11	17.66*	213.34	16.92*	530.23
**Average**	443.95	444.69	16.99	17.66*	213.26	16.92*	530.23
**Total Uncertainty**	0.102	0.107	0.108	0.100*	0.103	0.100*	0.102

Table S8: Binding energy values (in eV) for selected peaks from the spectra shown in Figure S5. Binding energy values were calculated by correcting the carbon 1s emission ascribable to adventitious C to 285.00 eV and shifting all other binding energies by the same amount. Values are reported to two decimal places, except for 'Total Uncertainty' values, which are reported to three decimal places. The 'Average' and 'Total Uncertainty' values were calculated before rounding. (*) Values for the In_{ox} $4d_{5/2}$ binding energy were constrained between 17.42 and 17.66 during fitting, and optimal overall fits have the In_{ox} $4d_{5/2}$ at the edge of its position constraints. Hf $4f_{7/2}$ binding energies were constrained between 16.82 and 16.92 during fitting, and optimal overall fits have the Hf $4f_{7/2}$ at the edge of its position constraints.

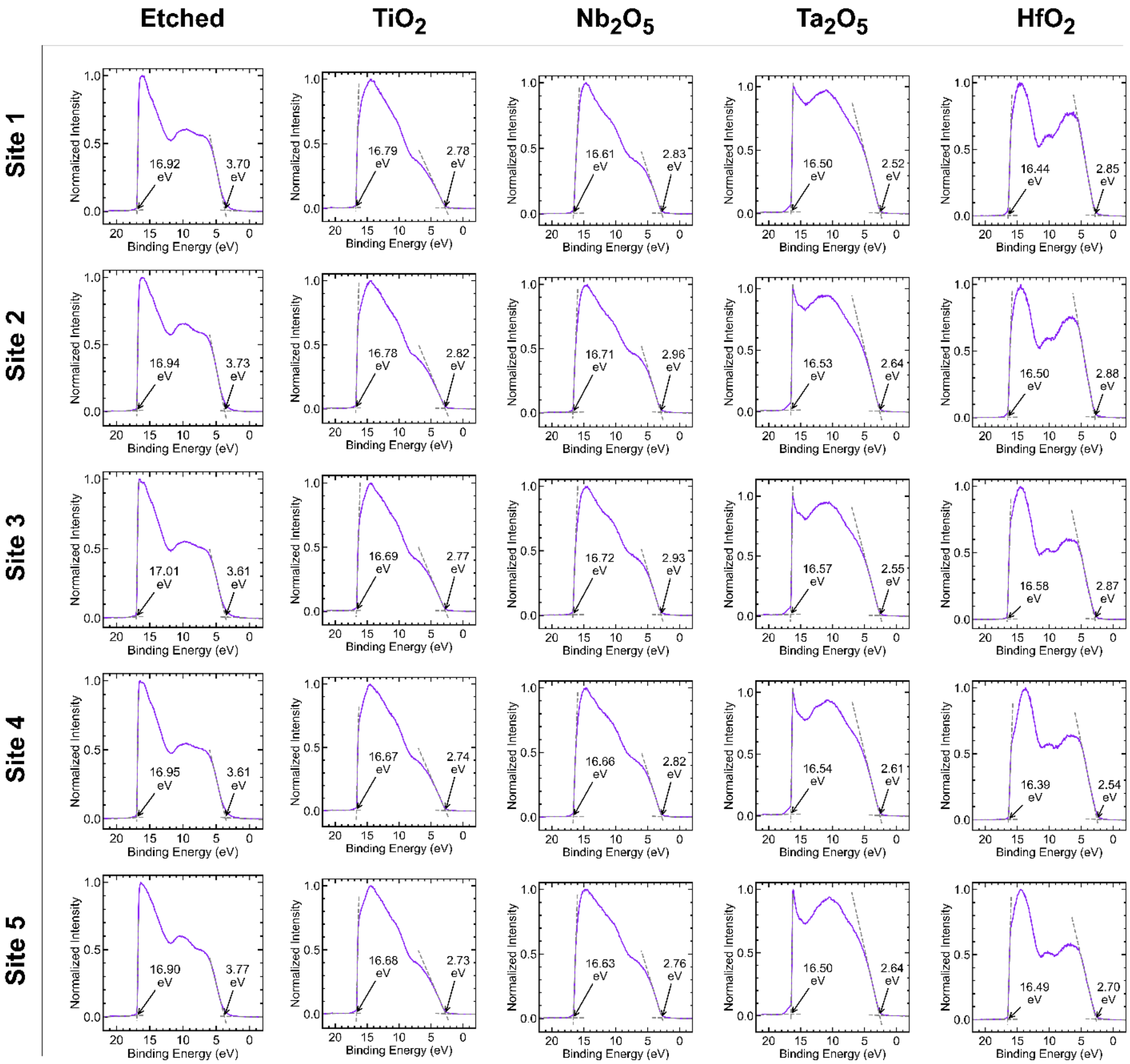

Figure S6: Replicate UP spectra from five replicate sites from etched p-InP and from the four metal oxide-coated p-InP samples used in this study.

Site	Etched	TiO_2-coated	Nb_2O_5-coated	Ta_2O_5-coated	HfO_2-coated
**1**	4.28	4.41	4.59	4.70	4.76
**2**	4.26	4.42	4.49	4.67	4.70
**3**	4.19	4.51	4.48	4.63	4.62
**4**	4.25	4.53	4.54	4.66	4.81
**5**	4.30	4.52	4.57	4.70	4.71
**Average**	4.26	4.48	4.53	4.67	4.72
**Total Uncertainty**	0.102	0.103	0.102	0.101	0.105

Table S9: Work function values (in eV) for samples from the spectra shown in Figure S6.

Site	Etched	TiO_2-coated	Nb_2O_5-coated	Ta_2O_5-coated	HfO_2-coated
**1**	3.70	2.78	2.83	2.52	2.85
**2**	3.73	2.82	2.96	2.64	2.88
**3**	3.61	2.77	2.93	2.55	2.87
**4**	3.61	2.74	2.82	2.61	2.54
**5**	3.77	2.73	2.76	2.64	2.70
**Average**	3.68	2.77	2.86	2.59	2.77
**Total Uncertainty**	0.105	0.101	0.107	0.103	0.120

Table S10: Energy values for the difference between the valence band maximum and the Fermi level (in eV) for samples from the spectra shown in Figure S6.

5. Construction of Band Diagrams

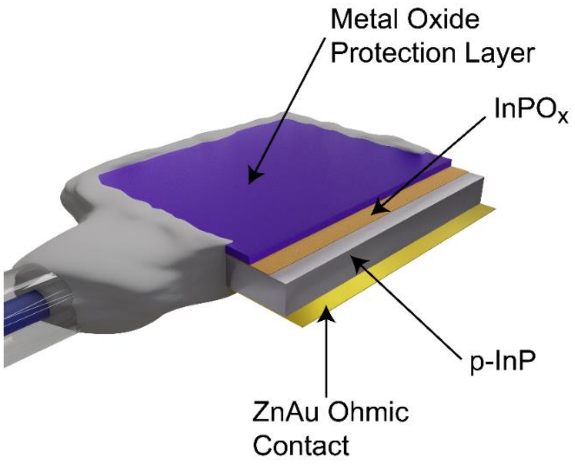

Figure S7: Diagram of photocathode components

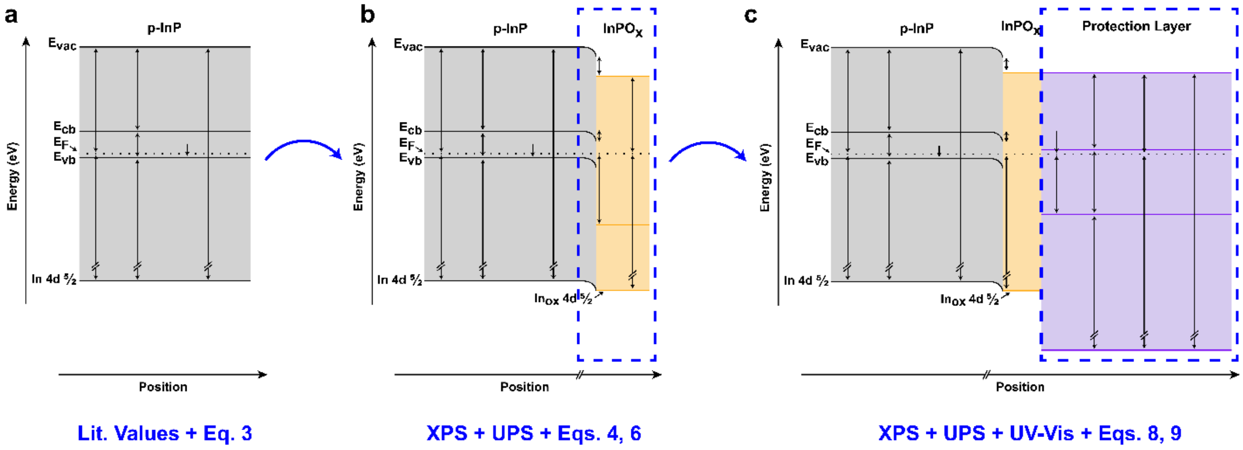

Figure S8: Stepwise construction of band diagrams. Figure S8a depicts the bulk InP. Figure S8b depicts the InP surface, along with its oxide layer. Figure S8c depicts the metal oxide protection layer.

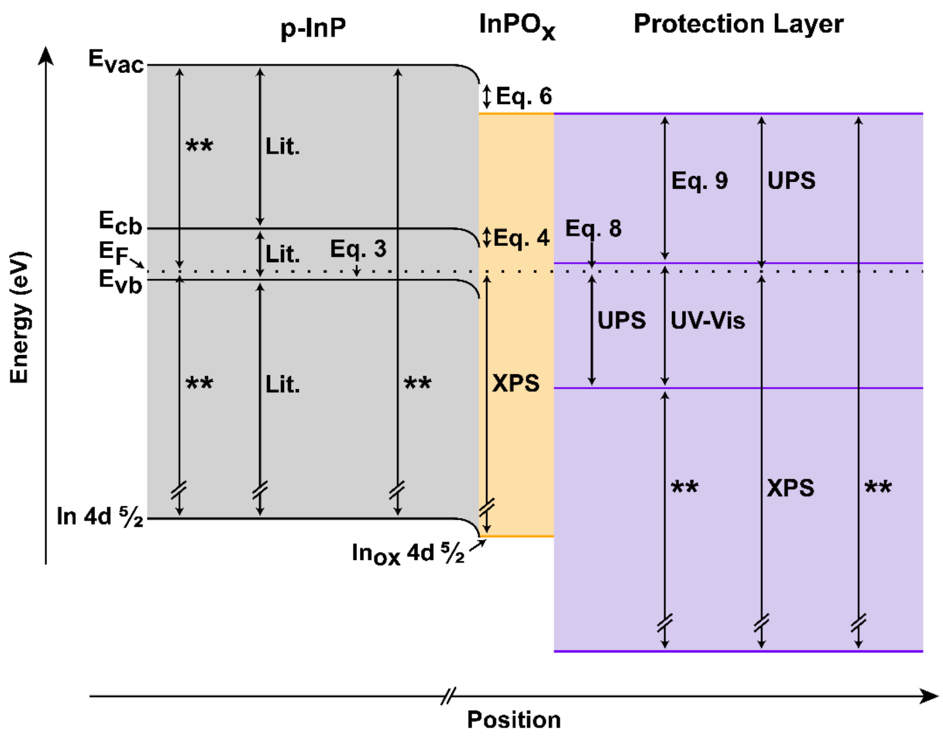

Figure S9: Sources for values in the band diagrams. 'Lit.' indicates a value taken from a literature source. '**' indicates a value calculated from other values on the diagram.

6. Surface Characterization of $InPO_x$

An etched p-InP sample was prepared as described in the Experimental Methods section of the main text and was immediately transferred to the load lock of the XPS and held under vacuum (<1-min exposure to air). Angle-resolved XPS was used to determine the surface composition of the sample at various depths. The electron take-off angle θ was defined as the angle between the sample surface normal and the photoelectron trajectory into the analyzer. Spectra were collected at θ=0°,55°,65°,75°, and 85°. Here, θ=0° corresponds to the conventional XPS geometry, whereas larger θ values approach grazing emission and thus yield increased surface sensitivity. Surface composition ratios of the etched p-InP wafer are reported in Table S11 and Figures S10-S12.

θ	In:P (%, total)	In_{InP}:In_{ox} (%)	P_{InP}:P_{ox} (%)
**0°**	49.23 : 50.77	90.09 : 9.91	88.61 : 11.39
**55°**	48.37 : 51.63	84.64 : 15.36	79.91 : 20.09
**65°**	48.76 : 51.24	84.88 : 15.12	78.72 : 21.28
**75°**	61.96 : 38.04	83.17 : 16.83	65.86 : 34.14
**85°**	82.36 : 17.64	79.07 : 20.93	Signal too low

Table S11: Angle-resolved XPS percent splits for etched p-InP (corrected area peaks). In:P (%) is referenced to the sum of the areas of the corrected In + P signals. In_{InP}:In_{ox} and P_{InP}:P_{ox} are percent splits of the total In and total P signals, respectively; each pair sums to 100% at a given angle.

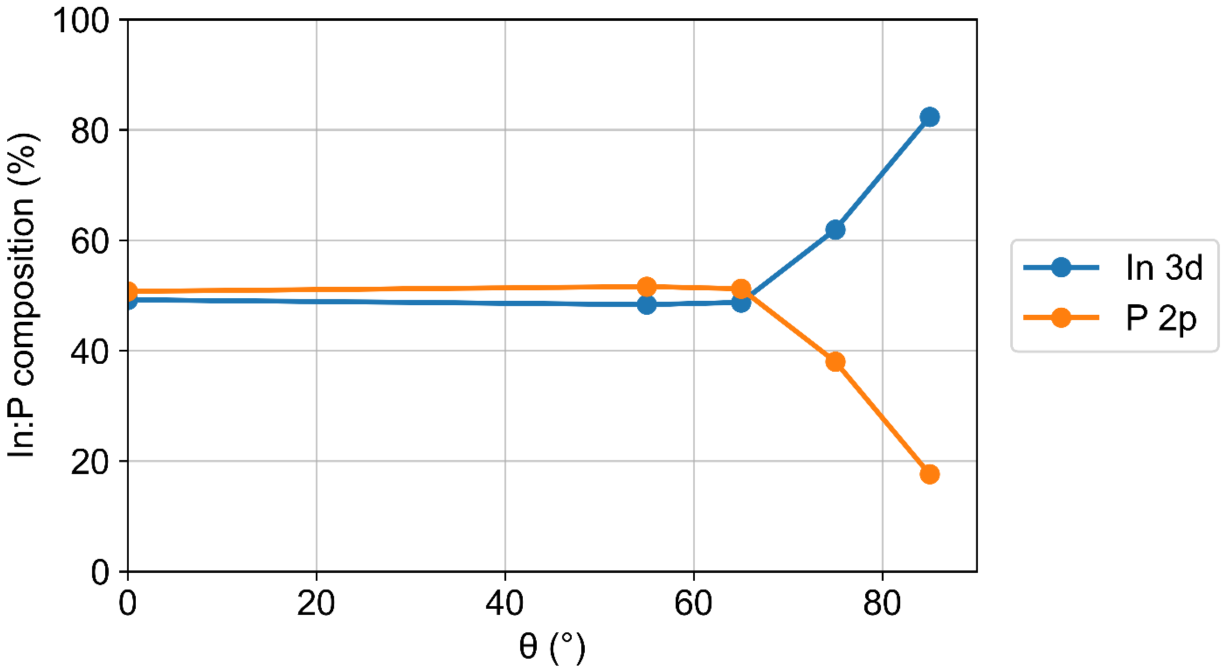

Figure S10: In:P (%) vs take-off angle θ.

Photoelectrons from different core levels have different mean escape depths (MEDs) because their kinetic energies and effective attenuation lengths (EALs) differ. For each emission angle and core level, we estimate the MED along the surface normal using equation S7 [refs.[4–6]]:

$$MED(\theta) \approx EAL(\theta) * \cos(\theta) \quad (S7)$$

Because $EAL_{P\ 2p} > EAL_{In\ 3d}$ at every angle θ, $MED_{P\ 2p} > MED_{In\ 3d}$ at every angle θ (Table S12, Figure S11).

	**Mean Escape Depth (nm)**	
θ	**In 3d**	**P 2p**
**0°**	1.7613	2.2123
**55°**	1.0277	1.2847
**65°**	0.7827	0.9682
**75°**	0.5539	0.6513
**85°**	0.4649	0.4910

Table S12: Photoelectron MEDs in nm per core level for all take-off angles used in this study.

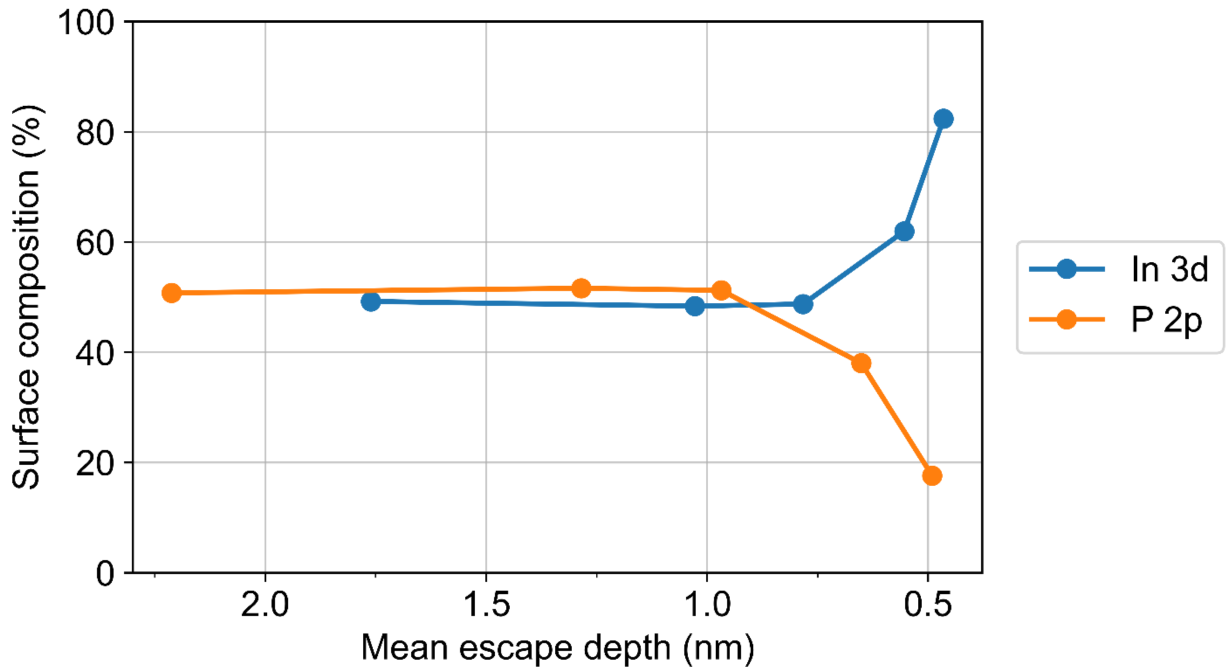

Figure S11: In:P (%) vs mean escape depth (MED).

Figure S12 shows the In:P (%) vs the MED, restricted to the common overlap in MED between In 3d and P 2p photoelectrons. In and P were linearly interpolated and renormalized so %In+%P = 100% at each MED. These values represent an exponentially weighted average of signal measured from the near-surface region, not point compositions at a specific depth from the surface. For example, at MED = 0.5 nm, the interpolated In:P ratio is 79.82% to 20.18%. Although this value does not represent the exact composition at a sharp plane 0.5 nm from the surface, a spectrum acquired at MED = 0.5 nm is dominated by signal from depths within ~0-1.5 nm of the surface (because ~95% of the signal is from < 3 * MED from the surface) and thus the interpolation approximates the surface composition at that scale.

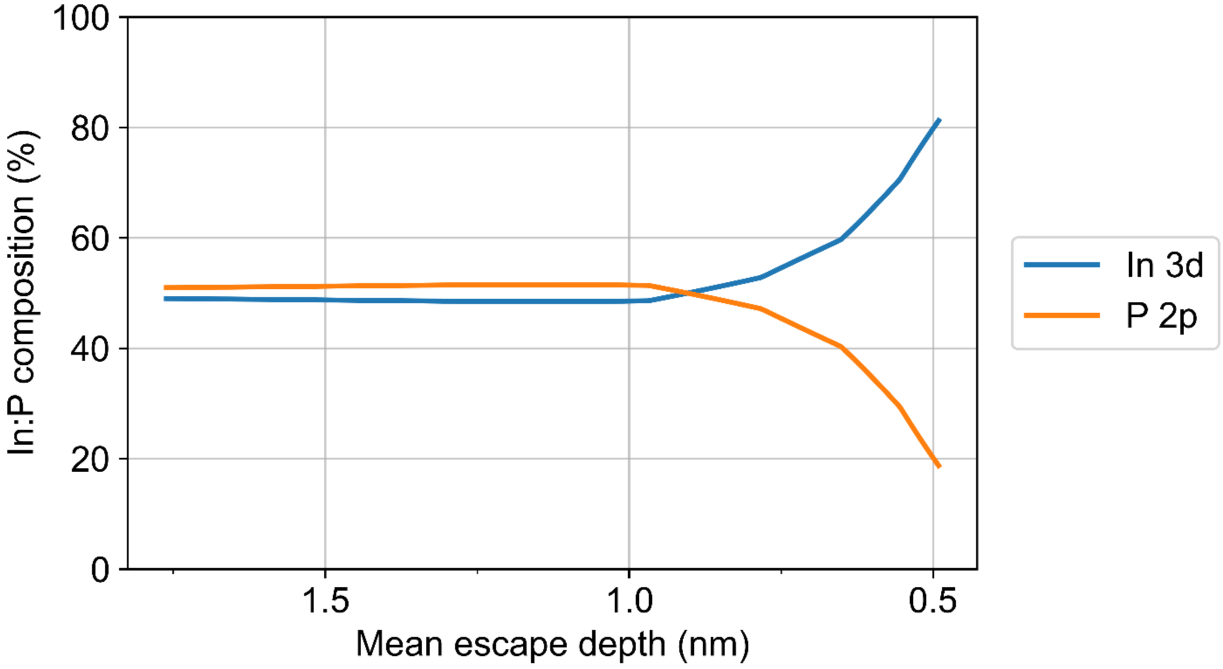

Figure S12: In:P (%) vs mean escape depth, restricted to the common overlap of MEDs for In 3d and P 2p, renormalized so the sum at each MED equals 100%.

Because the In_{InP} 3d and In_{ox} 3d components have nearly the same kinetic energy and thus the same EAL at a given angle, they have the same MED; likewise for P_{InP} and P_{ox}. Figures S13 and S14 show the ratio of In_{InP} to In_{ox} and the ratio of P_{InP} to P_{ox}, respectively.

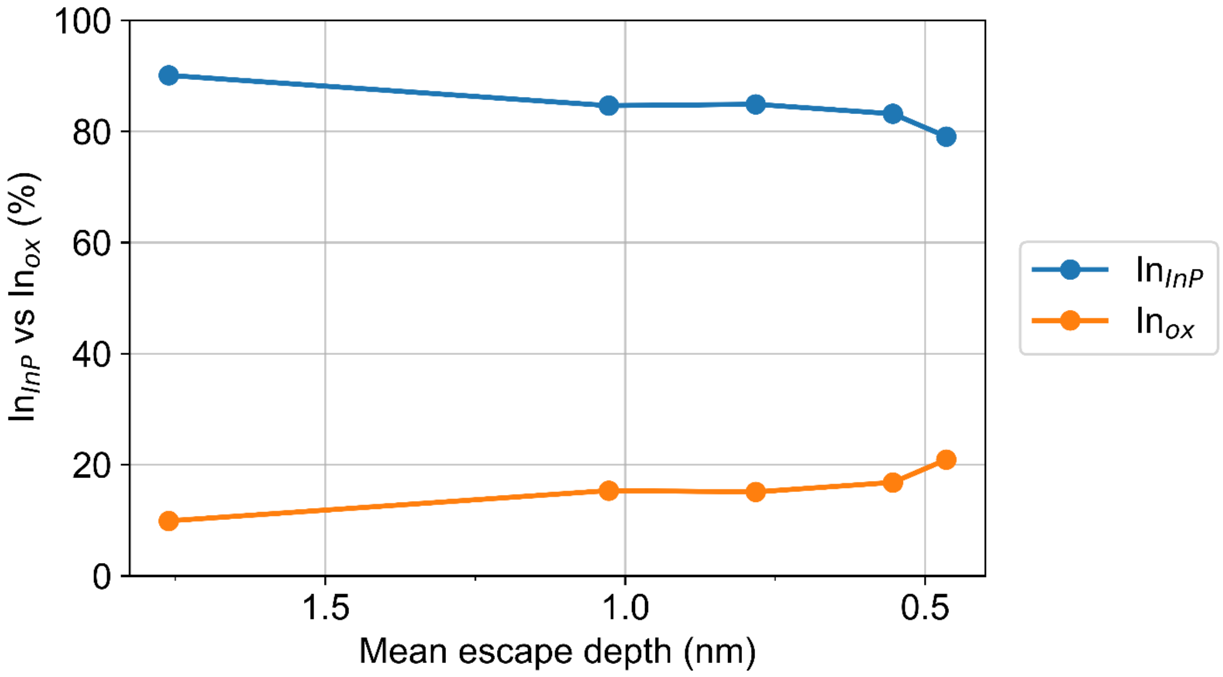

Figure S13: Ratio of In$_{Inp}$ to In$_{ox}$.

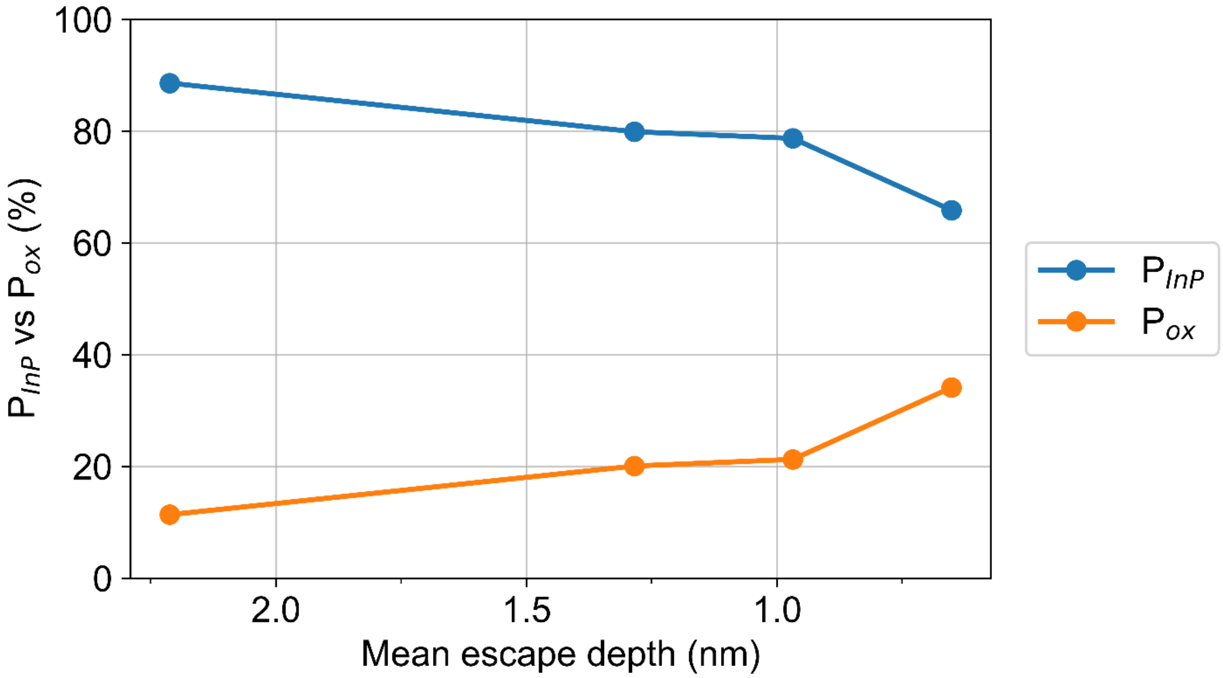

Figure S14: Ratio of P$_{Inp}$ to P$_{ox}$. Data points at MED = 0.4910 nm (θ = 85°) were not calculated because the small P signal was obscured by noise in the data.

Relative to the bulk, these data show that the surface of etched p-InP is depleted in P and enriched in In. Components of both In and P attributable to the oxide increased toward the surface, consistent with a non-uniform, In-rich surface oxide, as expected.[7–11]

The full In:P:O stoichiometry was not determined because the O 1s signal contains confounding contributions from physisorbed water and adventitious C-bound oxygen (C-O, C=O, O-C=O), some of which may also coordinate to the In oxide. Moreover, determination of the binding energy alone does not allow unambiguous assignment of phases in the $InPO_x$ system. The reported energies of the O 1s signals are very similar for many of the likely surface species. For instance, the O 1s binding energy of $In(OH)_3$ is reported as 531.2 eV, which is also the reported binding energy of In_2O_3. The O 1s component of $InPO_4$ has a binding energy of 531.3 eV[1].

Calculation of In:P Composition

Peak fitting was performed on the In 3d and P 2p regions of the XPS data. Background-subtracted peak areas were corrected using the relative sensitivity factor (RSF, Table S13), the analyzer transmission function, and the angle-dependent EAL to account for the sampling geometry. Additionally, the angle between the source and analyzer was updated in CasaXPS as 60° [ref.[12]]. Corrected In and P areas were then compared at each θ to obtain the In:P ratio reported in Table S11 and Figures S10-S12.

**Region**	**RSF**
**In 3d**	22.54
**P 2p**	1.192

Table S13: RSFs used for In:P (%).

EAL Calculations and Quantification of Inputs

The effective attenuation length (EAL) is the exponential attenuation parameter describing the decay of photoelectron intensity with depth for a given experimental geometry. At a depth equal to the EAL, the detected intensity is reduced to 1/e of its surface value. The EAL incorporates the effects of both inelastic and elastic scattering, as well as the emission angle. In contrast, the inelastic mean free path (IMFP) describes only the average distance an electron travels between successive inelastic scattering events and does not account for elastic scattering or measurement geometry.[13] EALs were computed using the NIST Electron Effective-Attenuation-Length Database.[14] EALs were calculated for photoelectrons in stoichiometric $InPO_4$ because the calculator requires inputs such as the material density and the number of valence electrons per molecule. Table S14 provides the calculated EAL values for In 3d and P 2p photoelectrons at each of the angles used in this study.

	**Effective Attenuation Length (nm)**	
θ	**In 3d**	**P 2p**
**0°**	1.7613	2.2123
**55°**	1.7918	2.2398
**65°**	1.8520	2.2909
**75°**	2.1400	2.5165
**85°**	5.3341	5.6331

Table S14: Effective attenuation length (EAL, nm) per core level and take-off angle.

Calculator inputs:

- **Class of material:** Inorganic compound
- **Technique:** XPS
- **Source of IMFP and TMFP values:** Database
- **Stoichiometry:** In = 1, P = 1, O = 4
- **Number of valence electrons per molecule:** 32
- **Band gap:** 4.5 eV [ref.[15]]
- **Predictive formula**: TPP-2M equation
- **Density:** 4.9 g/cm^3 [ref.[16]]
- **EAL type:** Practical
- **EAL definition:** Thickness measurement of an overlayer film
- **Electron kinetic energy:** See Table S15

- **Asymmetry parameter:** See Table S15
- **Experimental configuration:** See Table S16

Region	Electron Kinetic Energy (eV)	Asymmetry Parameter
**In 3d**	1042.5	1.22
**P 2p**	1357.6	1.10

Table S15: Parameters used per energy region to calculate EALs. Electron kinetic energies were calculated as 1486.6 eV (monochromated Al Kα) minus the binding energy of the dominant spin-orbit component (i.e. In 3d $\frac{5}{2}$ instead of In 3d $\frac{3}{2}$). Asymmetry parameters were taken from Table A.1. of the NIST Electron EAL Database Users' Guide.[14]

θ	X-ray incidence angle	Electron emission angle
**0°**	60°	0°
**55°**	5°	55°
**65°**	-5°	65°
**75°**	-15°	75°
**85°**	-25°	85°

Table S16: Parameters used in the 'Experimental Configuration' tab of the calculator.

7. Deconvolution of Hf 4f and In 4d Spectra

In the p-InP│interfacial oxide│HfO_2 system, the Hf 4f and In 4d envelopes overlap in binding energy, so deconvolution was required to determine the binding energy of the In_{InP} $4d_{5/2}$ peak. The deconvolution used three prior inputs: (i) a reference HfO_2 Hf $4f_{7/2}$ binding energy, (ii) the Hf:In composition for each spectrum (from non-overlapping core levels), and (iii) the In_{InP}:In_{ox} ratio in the sample (assessed from the In 3d core level).

Hf $4f_{7/2}$ Binding Energy Measurement

The Hf $4f_{7/2}$ binding energy was measured from an ALD HfO_2 film grown on a Cu foil, using the same ALD parameters as in the main text. Measurements on five locations on the Cu│HfO_2 sample yielded a mean Hf $4f_{7/2}$ binding energy of 16.86 eV (Figure S15, Table S17). Values were calibrated by setting adventitious C 1s to 285.00 eV and shifting all peaks accordingly.

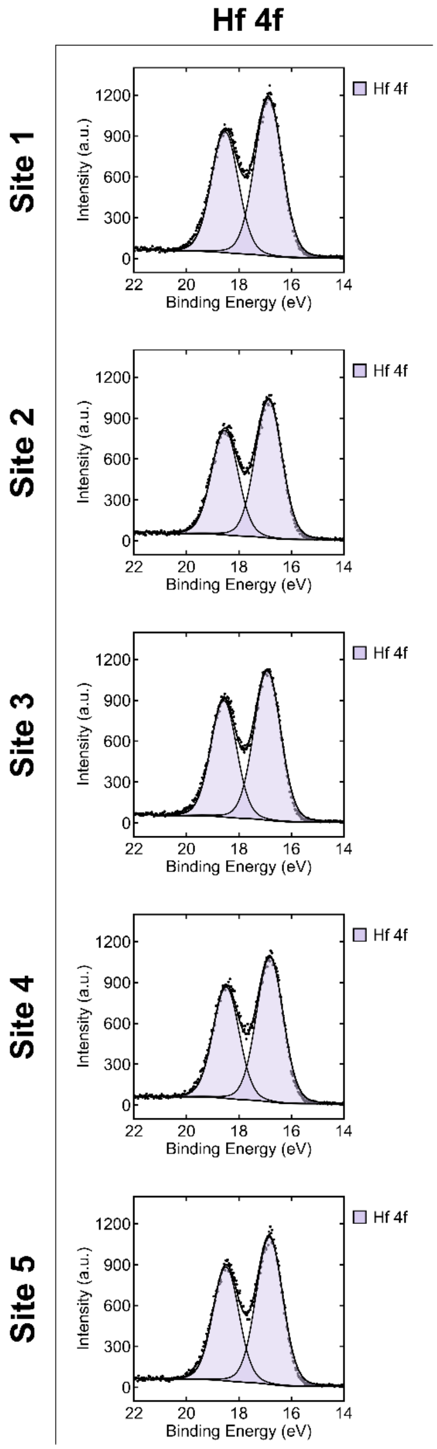

Figure S15: Replicate XP spectra from five separate locations on an HfO_2-coated Cu substrate.

Site	Hf $4f_{7/2}$
**1**	16.87
**2**	16.87
**3**	16.92
**4**	16.82
**5**	16.83
**Average**	16.86
**Total Uncertainty**	0.101

Table S17: Hf $4f_{7/2}$ binding energies (eV) from the spectra in Figure S15. Binding energy values were calibrated by setting the adventitious carbon 1s peak to 285.00 eV and shifting all other binding energies by the same amount. Values are reported to two decimal places, except for 'Total Uncertainty' values, which are reported to three decimal places. The 'Average' and 'Total Uncertainty' values were calculated before rounding.

Compositional Analyses

The Hf:In fraction for each p-InP│HfO_2 spectrum was obtained from the peak areas of the Hf 4d and In 3d emissions, which do not overlap. Background-subtracted areas were corrected for RSF (Table S18), the analyzer transmission function, and EAL (Table S22). The source-analyzer angle in CasaXPS was set to 60°. Table S19 presents the resulting percentage splits.

Region	RSF
**Hf 4d (total)**	14.97
**Hf 4f (total)**	7.52
**Hf $4f_{7/2}$**	4.20
**Hf $4f_{5/2}$**	3.32
**In 3d (total)**	22.54
**In 4d (total)**	2.28
**In $4d_{5/2}$**	1.365
**In $4d_{3/2}$**	0.91

Table S18: RSFs used for Hf and In quantification.

Site	Hf:In (%)
**1**	71.88 : 28.12
**2**	71.18 : 28.82
**3**	61.84 : 38.16
**4**	69.90 : 30.10
**5**	68.00 : 32.00

Table S19: Percent splits of Hf:In composition from Hf 4d and In 3d.

The In_{InP} 4d to In_{ox} 4d ratio used in the convoluted spectra was assumed to be the same as the ratio of In_{InP} $3d_{5/2}$ to In_{ox} $3d_{5/2}$ (Table S20).

Site	In_{InP} $3d_{5/2}$: In_{ox} $3d_{5/2}$ (%)
**1**	58.35 : 41.65
**2**	53.39 : 46.61
**3**	60.46 : 39.54
**4**	57.14 : 42.86
**5**	55.71 : 44.29

Table S20: Percent splits of In_{InP} $3d_{5/2}$ to In_{ox} $3d_{5/2}$ composition.

Peak Fitting Constraints

Deconvolution proceeded by first placing the Hf 4f doublet, then constraining the In 4d doublet areas based on the measured Hf:In and In_{InP}:In_{ox} fractions. Table S21 presents the plotting constraints for all peaks in the convoluted region.

The position and FWHM of the Hf $4f_{7/2}$ peaks were constrained based on the range of Hf $4f_{7/2}$ peak positions and FWHMs in the HfO_2 on Cu sample. The position and FWHM of the Hf $4f_{5/2}$ peaks were constrained based on the range of Hf $4f_{5/2}$ peak positions and FWHMs in the HfO_2 on Cu sample. The area of the Hf $4f_{5/2}$ peak was constrained to be 0.75 times the area of the Hf $4f_{7/2}$ peak, (spin-orbit ratio for 4f).

The area of the In_{InP} $4d_{5/2}$ peak was constrained in relation to the Hf $4f_{7/2}$ peak, and calculated as:

$$A(In_{InP}\ 4d_{5/2}) = A(Hf\ 4f_{7/2}) * \frac{\%\ In}{\%\ Hf} * \frac{RSF_{In\ 4d_{5/2}}}{RSF_{\ Hf\ 4f_{7/2}}} * f_{In_{InP}} \quad (S8)$$

where $\frac{\%\ In}{\%\ Hf}$ is from Table S19 and $f_{In_{InP}}$ is the fraction of total In signal assigned to InP (as opposed to oxide) from Table S20 (as a unit fraction).

The area of the In_{ox} $4d_{5/2}$ peak was analogously calculated as:

$$A(In_{ox}\ 4d_{5/2}) = A(Hf\ 4f_{7/2}) * \frac{\%\ In}{\%\ Hf} * \frac{RSF_{In\ 4d_{5/2}}}{RSF_{\ Hf\ 4f_{7/2}}} * f_{In_{ox}} \quad (S9)$$

The area of the In_{InP} $4d_{3/2}$ peak was constrained as 0.667 * In_{InP} $4d_{5/2}$ area. The area of the In_{ox} $4d_{3/2}$ peak was constrained as 0.667 * In_{ox} $4d_{5/2}$ area. FWHM constraints for all In 4d peaks were constrained based on the range of the FWHMs for the In 4d peaks from the Ta_2O_5 on p-InP spectra, as was the position of the In_{ox} $4d_{5/2}$ peak. The positions of the In_{InP} $4d_{5/2}$ and In_{InP} $4d_{3/2}$ peaks were unconstrained, and the position of the In_{ox} $4d_{3/2}$ was constrained as the position of the In_{ox} $4d_{5/2}$ + 0.90 eV.

Peak	Area Constraint	FWHM Constraint (min, max)	Position Constraint (eV)
**Hf $4f_{7/2}$**	—	1.13, 1.23	16.82, 16.92
**Hf $4f_{5/2}$**	0.75 * Hf $4f_{7/2}$	1.15, 1.20	18.48, 18.58
**In_{InP} $4d_{5/2}$**	Eq. S8	0.56, 0.62	—
**In_{ox} $4d_{5/2}$**	Eq. S9	0.73, 0.82	17.42, 17.66
**In_{InP} $4d_{3/2}$**	0.667 * In_{InP} $4d_{5/2}$	0.56, 0.64	—
**In_{ox} $4d_{3/2}$**	0.667 * In_{ox} $4d_{5/2}$	0.69, 0.97	In_{ox} $4d_{5/2}$ + 0.90

Table S21: Constraints per peak of the convoluted Hf 4f/In 4d spectra. '—' indicates no constraint imposed.

EAL Calculations and Quantification Inputs

EALs were computed using the NIST Electron EAL Database.[14] Table S22 presents the calculated EAL for In 3d and Hf 4d photoelectrons.

Region	Electron Kinetic Energy (eV)	Asymmetry Parameter	Effective Attenuation Length (nm)
**In 3d**	1042.5	1.22	1.3787
**Hf 4d**	1273.4	1.29	1.6250

Table S22: Electron kinetic energy (eV), asymmetry parameter, and effective attenuation length (EAL, nm) per core level. Electron kinetic energies were calculated as 1486.6 eV (monochromated Al Kα) minus the binding energy of the dominant spin-orbit component (i.e. In $3d_{5/2}$ instead of In $3d_{3/2}$). Asymmetry parameters were taken from Table A.1. of the NIST Electron EAL Database Users' Guide.[14]

Calculator inputs:

- **Class of material:** Inorganic compound
- **Technique:** XPS
- **Source of IMFP and TMFP values:** Database
- **Stoichiometry:** Hf = 1, O = 2
- **Number of valence electrons per molecule:** 16
- **Band gap:** 5.61 eV
- **Predictive formula**: TPP-2M equation
- **Density:** 9.7 g/cm^3 [ref.[17]]
- **EAL type:** Practical
- **EAL definition:** Thickness measurement of an overlayer film
- **Electron kinetic energy:** See Table S22
- **Asymmetry parameter:** See Table S22
- **Experimental configuration:** X-ray incidence angle = 60°, electron emission angle = 0°

8. References

(1) Henderson, J. D.; Pearson, L.; Nie, H.-Y.; Biesinger, M. C. X-Ray Photoelectron Spectroscopy Analysis of Indium and Indium-Containing Compounds. *Surf. Interface Anal.* **2024**, *57* (1), 81–97. https://doi.org/10.1002/sia.7356.

(2) Hu, S.; Richter, M. H.; Lichterman, M. F.; Beardslee, J.; Mayer, T.; Brunschwig, B. S.; Lewis, N. S. Electrical, Photoelectrochemical, and Photoelectron Spectroscopic Investigation of the Interfacial Transport and Energetics of Amorphous TiO_2/Si Heterojunctions. *J. Phys. Chem. C* **2016**, *120* (6), 3117–3129. https://doi.org/10.1021/acs.jpcc.5b09121.

(3) Kamptner, A.; Scharber, M. C.; Schiek, M. Accurate Determination of the Uniaxial Complex Refractive Index and the Optical Band Gap of Polymer Thin Films to Correlate Their Absorption Strength and Onset of Absorption. *ChemPhysChem* **2024**, *25* (23), e202400233. https://doi.org/10.1002/cphc.202400233.

(4) Jablonski, A.; Powell, C. J. Relationships between Electron Inelastic Mean Free Paths, Effective Attenuation Lengths, and Mean Escape Depths. *J. Electron Spectrosc. Relat. Phenom.* **1999**, *100* (1), 137–160. https://doi.org/10.1016/S0368-2048(99)00044-4.

(5) Powell, C. J.; Jablonski, A. Surface Sensitivity of X-Ray Photoelectron Spectroscopy. *Nucl. Instrum. Methods Phys. Res., Sect. A* **2009**, *601* (1–2), 54–65. https://doi.org/10.1016/j.nima.2008.12.103.

(6) Jablonski, A.; Powell, C. J. Practical Expressions for the Mean Escape Depth, the Information Depth, and the Effective Attenuation Length in Auger-Electron Spectroscopy and X-Ray Photoelectron Spectroscopy. *J. Vac. Sci. Technol. A* **2009**, *27* (2), 253–261. https://doi.org/10.1116/1.3071947.

(7) Hofmann, A.; Streubel, P.; Meisel, A. XPS Investigation of Oxide Films on InP(100). *Surf. Interface Anal.* **1988**, *12* (5), 315–319. https://doi.org/10.1002/sia.740120508.

(8) Hollinger, G.; Bergignat, E.; Joseph, J.; Robach, Y. On the Nature of Oxides on InP Surfaces. *J. Vac. Sci. Technol. A* **1985**, *3* (6), 2082–2088. https://doi.org/10.1116/1.572928.

(9) Wager, J. F.; Ellsworth, D. L.; Goodnick, S. M.; Wilmsen, C. W. Composition and Thermal Stability of Thin Native Oxides on InP. *J. Vac. Sci. Technol.* **1981**, *19* (3), 513–518. https://doi.org/10.1116/1.571049.

(10) Kurth, E.; Reif, A.; Gottschalch, V.; Finster, J.; Butter, E. Chemical Etching and Polishing of InP. *Cryst. Res. Technol.* **1988**, *23* (1), 117–126. https://doi.org/10.1002/crat.2170230117.

(11) Schwartz, G. P.; Sunder, W. A.; Griffiths, J. E. The In-P-O Phase Diagram: Construction and Applications. *J. Electrochem. Soc.* **1982**, *129* (6), 1361-1367. https://doi.org/10.1149/1.2124151.

(12) *Quantification by XPS Illustrated Using Fused Silica*; Casa Software Ltd, 2019. http://www.casaxps.com/casaxps-training/bgn_course/Quantification%20by%20XPS%20Illustrate%20using%20Fused%20Silica.pdf.

(13) Jablonski, A.; Powell, C. J. Effective Attenuation Lengths for Quantitative Determination of Surface Composition by Auger-Electron Spectroscopy and X-Ray Photoelectron Spectroscopy. *J. Electron Spectrosc. Relat. Phenom.* **2017**, *218*, 1–12. https://doi.org/10.1016/j.elspec.2017.04.008.

(14) NIST Standard Reference Database 82. *NIST* **2010**. http://dx.doi.org/10.18434/T4MK5P.

(15) Wager, J. F.; Wilmsen, C. W.; Kazmerski, L. L. Estimation of the Band Gap of $InPO_4$. *Appl. Phys. Lett.* **1983**, *42* (7), 589–591. https://doi.org/10.1063/1.94003.

(16) *Indium Phosphate*. American Elements. https://www.americanelements.com/indium-phosphate-14693-82-4 (accessed 2025-10-01).

(17) *Hafnium Oxide*. American Elements. https://www.americanelements.com/hafnium-oxide-12055-23-1 (accessed 2025-10-02).
